# Effectiveness of sensory adaptive dental environments to reduce psychophysiology responses of dental anxiety and support positive behaviours in children and young adults with intellectual and developmental disabilities: a systematic review and meta-analyses

**DOI:** 10.1186/s12903-023-03445-6

**Published:** 2023-10-19

**Authors:** Kaitlyn Reynolds, Ritesh Chimoriya, Navira Chandio, Danielle Tracey, Archana Pradhan, Paul Fahey, Nicole Stormon, Amit Arora

**Affiliations:** 1 School of Health Sciences, Western Sydney University, Penrith, NSW 2751 Australia; 2 Health Equity Laboratory, Campbelltown, NSW 2560 Australia; 3 School of Medicine, Western Sydney University, Campbelltown, NSW 2560 Australia; 4 Philanthropy Nepal (Paropakari Nepal) Research Collaboration, Auburn, NSW 2144 Australia; 5 Translational Health Research Institute, Western Sydney University, Campbelltown, NSW 2560 Australia; 6 Centre for Educational Research, Western Sydney University, Kingswood, NSW 2747 Australia; 7 Sydney Dental School, The University of Sydney, Surry Hills, NSW 2010 Australia; 8 School of Dentistry, The University of Queensland, Herston, QLD 4006 Australia; 9 Discipline of Child and Adolescent Health, The Children’s Hospital at Westmead Clinical School, Faculty of Medicine and Health, The University of Sydney, Westmead, NSW 2145 Australia; 10 Oral Health Services, Sydney Local Health District and Sydney Dental Hospital, NSW Health, Surry Hills, NSW 2010 Australia

**Keywords:** Oral health, Children, Dentistry, Multisensory, Oral hygiene, Sensory adapted, Occupational therapy, Dental anxiety, Sensory processing, Developmental disability, Intellectual disability

## Abstract

**Background:**

People with Intellectual and developmental disabilities (IDDs) experience oral health inequality due to myriad of risk factors and complex needs. Sensory processing difficulties, maladaptive behaviours and dental anxiety contribute to difficulties in receiving preventive and routine dental treatments. This study aimed to systematically review the evidence on the effectiveness of sensory adaptive dental environments (SADE) for children and young adults (up to the ages 24 years) with IDD to address cooperation and dental anxiety.

**Methods:**

This review was reported according to The Preferred Reporting Items for Systematic Reviews and Meta-Analysis (PRISMA) guidelines. MEDLINE (Ovid), The Cochrane Library, Embase, Google Scholar, Web of Science and OT Seeker were searched using appropriate terms to identify Randomised Control Trails (RCTs) that matched inclusion criteria. Screening was conducted by two reviewers after de-duplication based on titles and abstracts followed by full text retrieval. Quality of the included studies was assessed using Cochrane Risk of Bias (ROB)-2 for crossover trials and data extracted by two reviewers. The details of the interventions and effectiveness were compared and discussed narratively, and comparable outcomes were included to meta-analyses using R software.

**Results:**

A total of 622 articles were identified and five articles met eligibility for inclusion. Three studies used multi-sensory adaptations and one used single sensory adaptation of music. Narrative synthesis showed some evidence of SADE reducing magnitude and duration, although, questionable for reducing the number of maladaptive behaviours. Two studies demonstrated conflicting evidence of the effect of SADE on cooperation. Three studies demonstrated significant positive impact of SADE on psychophysiological outcomes. Despite an overall tendency to favour SADE, no statistically significant difference of maladaptive behaviours was found between SADE and regular dental environment (RDE) (Standardised mean change (SMC) = 0.51; 95% Confidence Interval (CI) -0.20 to 1.22; *p* = 0.161). SADE was superior to RDE (SMC -0.66; 95% CI -1.01 to -0.30; *p* =  < 0.001) in reducing psychophysiological responses of dental anxiety.

**Conclusion:**

Current evidence suggests that adapting visual, tactile, and auditory aspects of the dental environment in a single or multi-sensory approach demonstrates small positive effects on psychophysiological responses and maladaptive behaviours of dental anxiety for people with IDD.

**Trial registration:**

The title of this review was registered with PROSPERO (CRD42022322083).

**Supplementary Information:**

The online version contains supplementary material available at 10.1186/s12903-023-03445-6.

## Background

Intellectual and developmental disabilities (IDD) are a group of conditions due to physical, learning, language, sensory, or behaviour impairments that are characterised by significant limitations both in intellectual functioning and adaptive behaviour as expressed in conceptual, social, and practical adaptive skills [1]. These conditions impact day-to-day functioning and include attention deficit hyperactivity disorder, autism spectrum disorder, cerebral palsy,, intellectual disability, down syndrome, Fragile X Syndrome learning disability, and other developmental delays as classified by American Psychiatric Association [2]. A high prevalence of IDD has been documented in United States in a systematic review by Anderson, Larson [3] with children yielding a prevalence of 69.9 per 1,000 and 41.0 per 1,000 for adults. This picture is not unique with several other studies documenting similar high prevalences in other countries such as India [4], Australia [5] and United Kingdom [6].

Oral health is fundamental to overall psychological and physiological health, wellbeing, and quality of life [7–9]. People with IDD are disproportionately vulnerable to poorer oral health than the typical developing population [10–15] due to myriad of risk factors contributing to their complex needs [14]. Individuals with IDD experience motor, perceptual, language, sensory, cognitive, and behavioural impairments which can create difficulties in undertaking oral hygiene behaviours [12, 16]. Parents and caregivers report difficulty in undertaking toothbrushing for people with IDD [14]. Poorer oral hygiene behaviours have been found to result in plaque stagnation, gingival inflammation, and an increased risk for dental caries [10–14].

Evidence links people with IDDs limited participation in routine and preventative dental services to environmental and individual factors [11, 17]. Dental practitioners experience difficulty in meeting individualised and complex needs of populations with IDDs [18, 19] that is associated with the over-stimulating physical environments [20], hyper-empathy, sensory integration (SI) issues, challenges with waiting room, [21], oral aversion [22], maladaptive behaviours, and limited knowledge and understanding of dental providers [23, 24].

Dental anxiety has been found to be linked towards poorer oral health care outcomes for the general population across multiple studies [25–27]. Dental anxiety is described as a psychophysiological state in which an individual experiences abnormal worry or fear of dental treatment [26]. Dental anxiety is exacerbated by the dental environment [25, 27–31] increasing maladaptive behaviour and psychological responses reducing compliance in dental procedures [32]. Dental anxiety is a common issue among children and young adults. A meta-analysis by Grisolia, dos Santos [33] reported the pooled prevalence as 23.9% (95% CI = 20.4—27.3) globally. Corresponding prevalence in pre-schoolers, schoolchildren, and adolescents respectively; 36.5%, 25.8%, and 13.3%. Two studies [32, 34] identified significant levels of dental anxiety for people with IDD and found it to be a major factor influencing oral health in relation to increased non-compliance and maladaptive behaviours. Fallea et al. [34] results show that individuals with a higher the level of IDDs with lower cognitive functioning exhibited a higher percentage and severity of dental anxiety.

Current evidence has linked SI difficulties for individuals with IDD to poorer oral health [11, 17, 21, 23]. SI is defined by Ayres [35] a neurological function that processes and organises sensory modality from one's own body and the environment for functional outputs to engage in activities of daily living effectively [35]. Ayres [35] SI theory is guided by two critical principles: “the brain is a self-organising system” and “intersensory integration is foundational to function”. Sensory processing issues occur due to difficulties in registering, modulating, and discriminating inputs that lead to maladaptive responses and also other motor or psychophysiological responses [36]. Evidence highlights SI difficulties intensifies maladaptive behaviours [37–40], consequently increasing non-compliance in dental appointments [29, 41]. Uncooperative behaviours are provoked by the increased sensory input of the dental environment including smells, touch (specifically oral) and loud noise [28].

Literature exploring individual in anxiety-provoking situations have used physiological outcomes to find valid and reliable measurements of anxiety [42–45]. It is known that anxiety leads to physiological changes due to activating of the autonomic and sympathetic systems leading to increases in heart rate, blood pressure, sweat gland activity, and respiration [46]. Additionally, evidence also supports behaviour as a suitable outcome when examining anxiety for individuals.

Multiple approaches exist to address these barriers including pharmacological sedation and non-pharmacological strategies such as general anaesthesia, sedation, desensitisation, papoose boards, behavioural and cognitive training, positive reinforcement, video-modelling, social stories and tell-show-do techniques [47]. Literature extensively explores sedation and general anaesthesia limited impact on children and young adults with IDD quality of life [48–52]. Pharmacological practices fail to address the underlying cause of the maladaptive behaviours [16, 53] and limit individuals personal freedom and participation [54]. A recent retrospective study compared the dental records of special care needs population who underwent dental treatment under general anaesthesia versus non-pharmacological approach [55]. The study concluded that special needs population treated under general anaesthesia had higher caries experience, definite negative behaviour and numerous treatment needs compared to the non-pharmacological group highlighting the justifiable use of general anaesthesia [55]. Further, the study authors found that special needs population in the general anaesthetic group had higher incidence of new carious lesions after 24 months whilst the non-pharmacological group had better recall rates [55]. This highlights that for the reduction of burden of oral diseases, there is a need to adopt additional measures to ensure individuals with IDD increase participation and involvement in regular dental treatment.

Papoose boards are globally controversial. A scoping review found American guidelines supporting use in a dental setting. Whilst other studies in United Kingdom, Israel and Australia exploring the ethical considerations of providing protective stabilisation including the restriction of movement and airways [56]. Yet additional evidence explores the benefits of papoose as a tool for providing tactile sensory input and subsequently having positive effects on anxiety throughout dental procedures [57].

Sensory adapted dental environments (SADE) have been thoroughly studies in people with IDDs. SADE uses a multisensory environment, “Snoezelen room”; a combination of mesmerising sound, good lighting, vibration, tactile sensation, and aroma [58]. The aim of implementing these sensory adaptions is to regulate sensory responses such as ‘flight or fight’ and facilitate in the reduction of associated maladaptive behaviours and anxiety [59, 60]. A large amount of evidence supports this approach for individuals with IDD to reduce maladaptive behaviours and promote regulation in multiple settings [61–66]. Studies that have researched sensory adapted dental environments (SADE) have shown significant improvement in cooperation and reduction in dental anxiety and associated behaviours for this population [59, 60, 67, 68].

There appears to be limited research on the effectiveness of SADE to address dental anxiety for children and young adults with IDD. The assessment of previous reviews identified various knowledge gaps and lack of high-quality synthesised evidence. Most studies focus broadly on non-pharmaceutical strategies in general therefore are inadequate to address current research question [47, 69]. Another systematic review by Ismail et al. [70] was conducted, focusing on SADE impact for children. However, this study poorly reported methods to replicate the study and population wasn’t specific in diagnosis including disabilities and typical developing population. Consequently, limiting the generalisability of findings to practice. Therefore, there is no synthesis of literature known that encompasses children to young adults with IDD. This proposed review is distinctive and necessary as it specifically looks at this population specifically children and young adults regarding the effectiveness of SADE to increase participation and manage psychophysiological and behaviour responses of dental anxiety. This is essential to increase evidence-based practice to influence greater oral health care outcomes for this population.

This review aims to address three research questions:

1. What are common sensory environmental strategies used to decrease maladaptive behaviours and psychophysiology responses of dental anxiety in children and young adults with IDD?
2. Is SADE effective to reduce dental anxiety (behaviour and psychophysiology) in children and young adults with IDD?
3. Do SADE increase children and young adults with IDD participation in oral health procedures?

## Methods

This systematic review has been reported according to “The Preferred Reporting Items for Systematic Reviews and Meta-Analysis (PRISMA) [71]. The protocol of this systematic review has been registered with PROSPERO International Prospective Register of Systematic Reviews (CRD42022322083) [72]. The protocol of this systematic review has been published [73]*.*

### Eligibility criteria

The inclusion and exclusion criteria was formulated based on the focused PICOS (Population Intervention Comparator Outcome and Study Design) framework [74], see Additional file 3.

### Types of participants

Studies that included children or young adults up to the ages of 24 years with a diagnosis of IDD. There was no restriction on type or severity of IDD diagnosis.

### Types of interventions

Any intervention that implemented a SADE either during the procedure or waiting room were eligible for inclusion. The interventions designed to modulate sensory sensitivities that targeted any of the senses; sound, sight, touch, taste, smell, vestibular (sense of head movement in space), interception (sensations in relation to physiological/physical condition of the body) and proprioception (sensations from muscles and joints). Studies could have implemented a single or a multi-sensory approach. These strategies included partially dimmed room with lighting effects, somatosensory stimuli, vibroacoustic, deep pressure or visual distraction. The studies involving dental procedure by using sedative techniques were excluded from the review. See Additional file 7  for description of intervention criteria.

### Types of comparators

This review considered studies that compared the intervention to control (no intervention), waitlist or usual care (regular dental environment).

### Types of outcome measures

The International Classification Of Functioning [mu] [75], the oral health framework adaptation by Faulks and colleagues [76] were used to categorise the primary outcomes.

This included participation restriction, as well as body structure and function.

The outcome, participation restriction and activity participation included participants cooperation and behaviour during the dental procedure. Examples of acceptable outcome measure include cooperation, participation, or compliance scores (Frankl score, children’s dental behaviour rating scale, negative behaviour checklist, or anxiety and cooperation scale), questionnaires or interviews of participants, dentists, or parents.

The outcome, body structure and function included psychophysiology and anxiety responses. Examples of acceptable outcome measure include electrodermal activity (EDA), oxygen saturation, respiratory rate, skin conductance, blood pressure, and heart rate.

### Types of studies

This review only considered Randomised Controlled Trials (RCTs) including crossover, parallel-group, cluster, and factorial design. Non-randomised study designs including pre-post study designs and non-experimental observational study designs were excluded from this review to increase confidence in results, minimise confounding factors impacting results and improve quality of findings. There was no restriction on language or date.

### Search strategy

The PICOS framework was used to formulate the initial search terms tabulated in a logic grid. A combination of Medical Subject Headings (MeSH) terms and keywords using Boolean operators, phrase searching, spelling variations, and truncation were devised to increase sensitivity and ensure satisfactory search retrieval. The search strategy was pre-tested in Medline (OVID) by two reviewers (KR and NC), in consultation from a Health Sciences Librarian (Table 1). Once the Medline search was finalised, the search was subsequently adapted to the syntax and subject headings of the other databases. Finally, a hand search of the reference lists of relevant studies that match inclusion criteria and previously published systematic reviews was conducted to identify further eligible studies.

**Table 1:** Medline (OVID) search strategy

**Search**	**Query**	**Records retrieved** ^a^
#1	(Child* or adolescen* or teen or youth or young adult or pe?diatric* or preschool or infant*).ti,ab	2,231,960
#2	Child, Preschool/ or Pediatrics/ or Adolescent/ or Child/ or infant/ or young adult/	4,006,547
#3	#1 or #2	4,667,012
#4	(Developmental disabilit* or intellectual disabilit* or special need* or mental retardation or disabl* or autis* or ADHD or ASD or Cerebral palsy or attention deficit hyperactivity disorder or Down Syndrome or Fragile X Syndrome or Fetal alcohol spectrum disorder).ti,ab	239,671
#5	Attention Deficit Disorder with Hyperactivity/ or Cerebral Palsy/ or Autistic Disorder/ or Disabled Persons/ or Disabled Children/ or child development disorders, pervasive/ or developmental disabilities/ or intellectual disability/ or Down Syndrome/ or Fragile X Syndrome/ or Fetal Alcohol Spectrum Disorders/	235,101
#6	#4 or #5	355,890
#7	#3 and #6	200,849
#8	((Dental adj3 (sensory adapted environment* or Multi-sensory environment*)) or Snoezelen or SADE).ti,ab	199
#9	Health facility environment/ or environment/ or dental offices/ or environmental adaption/ or Environment, Controlled/	78,822
#10	#8 or #9	78,997
#11	(((Oral or dental) adj (health or hygiene or anxiety)) or behaviour or compliance or physiological or pain or arousal or stress or psychological).ti,ab	2,606,469
#12	Stress, Psychological/ or Adaptation, Psychological/ or Psychological Distress/ or Arousal/ or Pain Perception/ or Pain/ or Dental Care/ or Dental Anxiety/ or Sensation/ or Patient Compliance/ or "Treatment Adherence and Compliance"/ or Oral Health/ or Oral hygiene/	512,327
#13	11 or 12	2,832,243
#14	7 and 10 and 13	161

^a^Medline (OVID) search was conducted on 18 August 2022

### Information sources

The following electronic databases were searched, without any restriction on publication date, type, language, or region: Medline (OVID), The Cochrane Library, Embase, Web of Science, OT seeker and Google Scholar (first 10 pages with 10 results per page totalling 100 results). The search was conducted on 14^th^ of March 2022 and then subsequently updated on the 18th of August 2022.

### Selection process

**Fig. 1:**
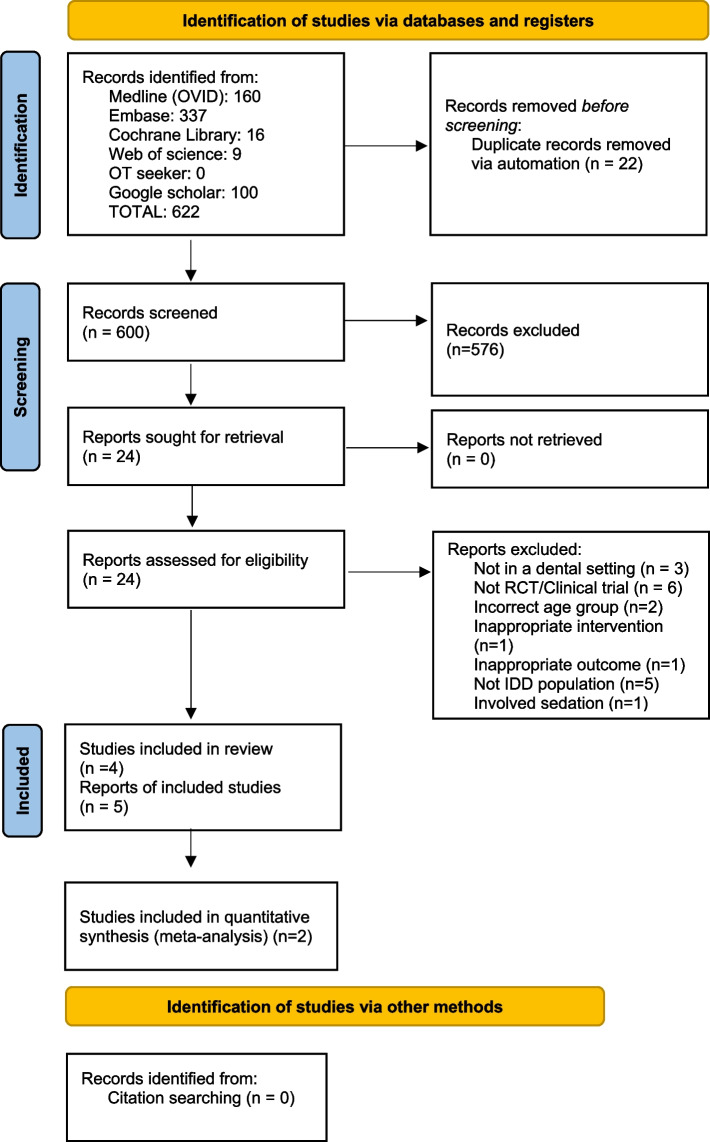
Preferred reporting items for systematic reviews and meta‐analyses flow diagram of literature search and study selection process

Studies identified via electronic databases and hand searching were imported into EndNote X9 [77] and duplicates removed. Following a pilot test, the title and abstracts of the studies were screened by two independent reviewers (KR and AA) against strict eligibility criteria, and if unclear, the full text was retrieved. Articles that met the inclusion criteria were retrieved and details recorded. The full text articles were reviewed by two reviewers (KR and AA) independently. When required, the study authors were contacted to seek additional information. A total of two contact attempts with the authors of the publication were made, and in case of no response, the article was screened based on the available information. Any disagreements that arose between the reviewers during the selection process was discussed with third reviewer (NC). Multiple published reports from a single study were analysed together. Throughout this process all reasons for exclusion of papers at the full text review stage was recorded see Additional file 5. The results of the study selection process was presented in a Preferred Reporting Items for Systematic Reviews and Meta-analyses (PRISMA) flow diagram (Fig. 1) [71].

### Data collection process and data items

A standardised data extraction form was developed, and pilot tested on one study. Subsequently the form was refined to ensure all relevant data captured. To ensure consistency across reviewers, a calibration exercise was undertaken. Two review authors (KR and AA) independently extracted data, discrepancies when identified were resolved through discussion with a third author (NC). Authors of papers were contacted to request missing or additional data, where required. A total of two contact attempts were made, and in case of no response, the study was assessed based on the available information. The data extracted was entered into an excel sheet including specific details about the study: article details, participant characteristics, intervention description, outcome measures and funding.

### Study risk of bias assessment

The Cochrane Risk of Bias (RoB)-2 tool for crossover trials [78] was used to assess the methodological quality of included studies. Each study was given a rating of high, low or some concerns in the following domains: randomisation process, period and carry-over effects, deviations from the intended interventions, missing outcome data, measurement of the outcome and selection of the reported result. Two reviewers (KR and RC) independently assessed the methodological quality of each study included in this review. Any disagreements that arose between the reviewers were resolved through discussion with a third reviewer (AA). The level of risk of bias in each of these domains were presented separately for each study in tables, figures and contextualised in a descriptive format. Study authors were contacted in the event of insufficient details being available to confidently assess the methodological quality; and if a response was not received after two attempts, the study quality was assessed based on the available information.

### Effect measures and synthesis methods

Meta-analyses were conducted to increase the precision and power of the intervention effects [79, 80]. All the statistical analyses were performed using the “metafor” package (2.0–0) of R statistical software (version 4.2.1) [81]. An assessment of the studies’ suitability for pooled analyses was made following the data extraction process. As all studies were cross-over design a moderate correlation of 0.5 was assumed and weighted or standardised final post-intervention mean differences (for continuous data) were used to calculate effect sizes, and their 95% confidence intervals (CI) were used for analyses. The studies were combined using a random effects model as significant heterogeneity was expected across studies (*p* ≤ 0.05 and/or I^2^ > 50%) [82]. All the statistical tests were two-sided, with a significance threshold of *p* < 0.05. This was based on the statistical guidelines of the Cochrane Handbook for Systematic Reviews of Interventions [83]. Between-study heterogeneity was evaluated using Cochran's Q test and quantified by the I^2^ statistic, with values ≥ 75% indicating high heterogeneity, 51–74% indicating moderate heterogeneity [84, 85]. The meta-analyses results are presented in a forest plot. A *priori* plan was to conduct sub-group analyses and use a funnel plot to evaluate publication bias with statistical tests of asymmetry, however, the review had insufficient data (under 10 studies). Where statistical pooling was not possible and due to substantial heterogeneity, a narrative synthesis of the study findings including tables was produced.

## Results

### Study selection

In total, 622 studies were identified across six databases. No further articles were retrieved through the comprehensive citation searching. After the duplicates were removed (*n* = 22), the titles and abstracts were reviewed by two independent reviewers (KR and AA) and a further 576 studies were excluded as they did not match the inclusion criteria. Twenty-four articles were assessed for eligibility based on full text review and only five articles were included in this review. Two papers reported on the same study and were combined for results, therefore there was a final total of four studies in this review. The excluded studies (*n* = 19) and the reasons for exclusion are summarised in Additional file 5. The PRISMA flow diagram [71] shows the identification, screening, eligibility, and included studies in Fig. 1.

### Study characteristics

The included studies were experimental randomised cross-over design that measured the effectiveness of SADE using the difference between two phases, SADE, and Regular dental environment (RDE) with 1- 4 months apart [60, 68, 86–88]. These studies were all conducted and published during 2009 to 2021 [60, 68, 86–88]. These studies had a sample size of 16–22 participants [60, 68, 86–88]. Two studies were conducted in America [60, 68], with the remaining studies conducted in India [88], and Israel [86, 87]. Table 2 summarises: (a) participant characteristics, (b) dental procedure completed, (c) SADE adaptions, (d) RDE description, (e) intervention outcomes, and (f) funding of the four included studies.

**Table 2:** Study characteristics summary of the included studies

Article details	Participant characteristics	Dental procedure completed	SADE adaptions		RDE description	Outcomes Measures	Funding
Kim et al., 2019 [60] USA Pilot experimental RCOT	**Sample size:** 22 **Sex**: 8F, 14 M **Age**: 16–21 years **Diagnosis**: Developmental disabilities including Down Syndrome, ASD, Cerebral palsy, developmental delay, intellectual, ADHD and others **Demographic:** High parent educational levels **Other:** 50% non-verbal. 48–72% with probable sensory modulation disorder and more than/much more than others in all sensory domain. 70% previously used general anaesthesia. 20% with history of papoose board	Routine dental exam and cleaning (no further detail)	Visual, auditory, and tactile		Fluorescent lighting (ceiling and overhead light) and without sensory stimuli adaptions	**Physiological**: heart rate and oxygen saturation (EDA) **Cooperation**: Frankl score	VCU Alexander Fellowship Grant
Shapiro et al., 2009 [86] Shapiro et al., 2009 [87] Israel Open RCOT	**Sample size:** 16 **Sex**: 11 M, 5F **Age**: 6–11 years **Diagnosis**: Developmental disabilities **Demographic**: no report **Other**: Autism excluded. Sever-moderate mental retardation. Parent reported anxious behaviours in all children	Dental scaling and polishing including manual cleaning of calcified deposits and brushing with a low-speed, power-driven dental handpiece with a rotary bristle brush	Visual, auditory, somato-sensory, and tactile		No sensory stimuli. Fluorescent lighting ceiling and overhead. Butterfly wrap less tight	**Physiological**: Standardized anxiety and cooperation scale and EDA **Behaviour**: Negative behaviours checklist Duration of negative behaviours	Lorraine White Trust
Cermak et al., 2015 [68] USA Experimental RCOT	**Sample size:** 22 **Sex**: 4F, 18 M **Age** 6–12 years **Diagnosis**: ASD **Demographic:** 96% Caucasian. 82% Hispanic, Latino. High maternal and parental education **Other:** 90% low-moderate communication (VABS-11). High sensory difficulties with 96% definite and 4.5 probable difference (SSP). Half with clinical dental anxiety (CFSS-DS)	Oral examination, prophylaxis, and fluoride application	Visual, auditory, and tactile		Standard manner. Without adaptions	**Physiological**: EDA **Behaviour**: CDBRS, Frankl scale **Pain:** FPS-R **Cooperation:** ACS, Dental sensitivity scale	National Institute of Dental and Craniofacial Research and seed grant from the Ostrow School of Dentistry and The Eunice Kennedy Shriver Institute of Child Health and Human Development
Gowdham et al. 2021 [88] India RCOT	**Sample size:** 20 **Sex**: Both genders but details not reported **Age** 6–14 years **Diagnosis**: Mild intellectual disability as specified by the Diagnostic and Statistical Manual of Mental Disorders–V **Demographic:** None reported **Other:** Hearing acuity within normal limits, I.Q. score between 50–69	Examination using dental hand instruments (mouth mirror and CPITN probe) Oral prophylaxis using an ultrasonic scaler Operative stimuli (auditory) using handpiece without bur		Auditory	Standard manner	**Physiological**: EDA	Not Reported

*SADE* Sensory adapted environments, *RDE* Regular dental environment, *EDA* Electrodermal Activity, *NS-SCRs* Non-specific skin conductance responses, *CDBRS* Children’s dental behaviour rating scale, *SCL* Skin conductance level, *NSSCR* Non-specific skin conductance responses, *DSSS* Sensory discomfort in the dental environment, *VABS-II* Vineland Adaptive Behaviour Scales II Expressive Language subtest, *SSP* Short sensory profile, *SADE* Sensory adapted environments, *RDE* Regular dental environment, *CASI-Anx* Child and Adolescent Symptom Inventory-Anxiety Scale, *CFSS-DS*, Children’s Fear Survey Schedule—Dental Subscale, *EDA* Electrodermal Activity CDBRS Children’s dental behaviour rating scale, *ACS* Anxiety cooperation scale, *FPS-R* Faces Pain scale-revisits, *TD* Typical developing, *ASD* Autism Spectrum Disorder, *F* Female, *M* Male, *EDA* Electrodermal Activity, *SADE* Sensory adapted dental environment, *RDE* Regular dental environment, *RCOT* Randomised cross-over trail, *RCOT* Randomised cross-over trail, *M* Male, *F* Female

### Participants

All included studies evaluated SADE for population with IDD [60, 68, 86–88]. The IDD diagnosis varied across studies. One study focused on the developmental disability of Autism specifically [68], two studies (with three published articles) reported on severe to moderate developmental disabilities [60, 86, 87] and another study sample was mild intellectual disabilities [88]. One study reported specific diagnosis including down syndrome, autism, cerebral palsy, developmental delay, intellectual, attention deficit hyperactivity disorder (ADHD) and others [60]. The study authors established the diagnoses using either a standardised measure or by relying on a diagnosis made by a paediatrician, psychiatrist, or clinical psychologist [60, 68, 86–88]. Participants had low language skills reported across two studies with 50% non-verbal [60] and 90% low -moderate communication [68]. The other two articles did not report on the type of developmental disabilities included within the study [86, 87]. Three studies focused on children aged 6–14 years [68, 86–88] and one study focused on adolescents and young adults aged 16–21 years [60].

The included studies where gender was reported, the total number of males with IDD was greater than the total number of females (total number = 17 females, 43males) [60, 68, 86, 87]. One study reported including male and females although, did not report the numerical details [88]. In two studies, SI difficulties were established, using short sensory profile with 48–72% probable [60] and 96% definite [68]. Dental anxiety was present across the studies with 70% participants previously using general anaesthesia and 20% with a history of papoose board [60], and apparent anxious behaviours in all children reported by their parents [86, 87]. Half of the participants had clinical dental anxiety reported using Children’s Fear Survey Schedule—Dental Subscale (CFSS-DS) [68]. Two studies reported high parent education levels [60, 68] and the additional studies failed to comment on further sociodemographic details.

### Outcomes

The outcomes in the included studies focus on psychophysiological responses of dental anxiety including heart rate, oxygen saturation [60], EDA [68, 86–88] and anxiety and cooperation scale [68]. Frankl scale [60, 68], the negative behaviour checklist [86, 87], duration of behaviours [86], anxiety cooperation scale, dental sensitivity scale and Children’s dental behaviour rating scale [68] was used to assess behaviour and cooperation.

### Interventions

All studies evaluated sensory adaptions within the dental environment specifically during routine oral examination and prophylaxis (teeth cleaning) [60, 68, 86–88]. In addition, the procedure of one study was fluoride application [60]. All SADE were compared to a RDE that consisted of no adaptions to sensory input [60, 68, 86–88]. Three studies evaluated multi-sensory adaptions including visual, tactile, and auditory [60, 68, 86, 87]. Only one study evaluated a single sensory approach using music exclusively [88].

### Common sensory adaptions used

Amongst the included studies similar sensory adaptions were implemented that included various visual, tactile, and auditory adaptions [60, 68, 86–88]. These adaptions are summarised in Table 3 for each study.

**Table 3:** Summary of the included studies intervention characteristics

Author	Visual	Tactile	Auditory	Additional aspects/comments
Kim et al., 2019 [60]	Solar projector and overhead light only	Regular X-ray lead apron (laid on patient)	Calming nature music	
Shapiro et al., 2009 [86]: Shapiro et al., 2009 [87]	• Removed all direct florescent lighting (50 Hz) including overhead lamp • Dimmed upward reflective fluorescent lighting (30–40,000 Hz) • Slow-moving repetitive visual colour effects via projector in child’s field • Dentist wore head-mounted narrow-spectrum light-emitting diode source lamp directly into child’s mouth	Butterfly wrap	Rhythmic music via loud (75 db)	• Bass vibrator (4 khms) on chair providing somatosensory stimulation • Butterfly wrap introduced to patients prior
Cermak et al., 2015 [68]	• Darkening curtains • All direct overhead fluorescent lighting and overhead lamp turned off • Lamp placed in back corner for ambient lighting • Slow moving visual colour effects onto ceiling (swimming fish or bubbles) • Dentist wore a head-mounted lamp directed into child’s mouth	Weighted butterfly wrap shoulders to ankles with dental X-ray vest	Rhythmic music via speakers	Prior social story for electrodes and sensory adaptions
Gowdham et al. 2021 [88]	N/A	N/A	Relaxing Indian instrumental music on phone handheld by operator and headphone provided to children to listen to the music	N/A

### Visual adaptation

The visual adaptions to the dental environment varied across studies with predominantly the reduction of light. This included the removal of all direct florescent lighting (including overhead dental lamp) that was replaced by direct head-mounted lamp into the child’s mouth by two articles [68, 86, 87]. Kim et al. [60] reduced lighting, however kept the overhead lighting. Cermak et al. [68] further reduced lighting with darkening curtains and using a lamp in back corner for ambient lighting. Three studies used slow moving colour effects projected within the visual field of the child [60, 68, 86, 87].

### Tactile adaptation

Kim et al. [60] used tactile adaptation of a regular x-ray lead apron laid across the child. Whereas, Cermak et al. [68] and Shapiro et al. [86, 87] used a weighted butterfly papoose that provided deep pressure to the shoulders and ankles. Only one study provided additional tactile input via somatosensory stimulation. This was received via bass vibrator (4khms) connected to the dental chair [86, 87].

### Auditory adaptation

Calming rhythmic nature music was played using speakers for all studies [60, 68, 86, 87] and specifically at 75db for one study [86, 87]. One study used headphones provided to the children that played relaxing Indian instrumental music [88].

### Effectiveness of SADE

Table 4 presents the results of the effectiveness of SADE from the included studies in this review.

**Table 4:** Summary of the included studies results

Author	Data analysis undertaken	Findings—raw data	Findings -Narrative/summary	Conclusions	Reviewers’ conclusions	Limitations
Kim et al., 2019 [60]	Paired analysis using Wilcoxon Signed Rank test for both visit subjects Repeated measures ANOVA models Post hoc pairwise comparisons were analysed using Tukey's HSD to adjust for multiple comparisons *P*-value 0.05 significant	**Cooperation/Behaviour** Frankl • M = 1 difference (4-point scale) towards SADE.* p* = 0.0703 clinician and *p* = 0.109 independent • ANOVA, observer Frankl scores were significantly higher with SADE setting than the RDE setting (average difference = 0.443; *p* = 0.037) and clinician's Frankl scores (average difference = 0.435; *p* = 0.018) • Repeated measures ANOVA, difference with verbal abilities (*p* = 0.044), age ( *p* = 0.084 with 2.4 6–10 vs 4.14 for 16–21 and 3.18 11–15 years) history of papoose (2.1 vs 3). None statistically significant Parent survey • 54% strongly agreed and 46% agreed that the SADE improved their child's dental anxiety • 46% strongly agreed, 36% agreed and 15% neutral that they would prefer the SADE for their child's next visit • 31% strongly agreed, 62% agreed and 8% neutral that SADE improved cooperation • 38% strongly agreed and 63% agreed better cooperation compared to previous **Physiological** Heart rate • Pre: similar (SADE 92, RDE 94, paired difference 0, *p* = 0.986) • Post: similar (SADE 100.5, RDE 99, paired difference -2, *p* = 0.814) Oxygen saturation • Pre: similar (SADE 0.99, RDE 0.99, paired difference 0, *p* = 1.000) • Post: similar (SADE 0.99, RDE 0.99, paired difference 0, *p* = 0.859)	**Cooperation/Behaviour** Frankl • Positive impact on behaviour with significantly higher scores. Paired analysis not significant but below 0.10 suggested for preliminary results Parent survey • All parents strongly agreed/agreed that SADE improved their child's dental anxiety • 82% strongly agreed/agreed they would prefer SADE for next visit • 94% strongly agreed/agreed SADE improved cooperation • All parents strongly agreed/agreed better cooperation compared to previous **Physiological** Heart rate: No change Oxygen saturation: No change	“SADE may be associated with improved behaviour in children with DD” (developmental disability) Clinical recommendation: “1. Pre-appointment sensory/behaviour assessment… 2. Tailoring the sensory modifications specific to child’s needs… 3. Future studies measure patient comfort”	Modifying sensory stimuli in a dental setting improves the dental experience of children with disabilities. Specifically improving behaviour and cooperation. However, no change in physiological factors (dental anxiety) was shown in study	1. Physiological measurement 2. Inability to blind raters to the treatment group 3. Parental bias 4. High loss to follow up (36/44)
Shapiro et al., 2009 [86]: Shapiro et al., 2009 [87]	2-tailed and p-value 0.05 significant ANOVA for treatment and sequence effect Paired t-tests compared degree of relaxation and arousal 2-tailed and p-value 0.05 significant	**Behaviours** • Number of anxious behaviours; SADE 4.06 and RDE 4.56. No significant effect; F-1.88, *p* = 0.19 • Magnitude of anxious behaviours; SADE 8.5 and RDE 15.5. Significant effect; F = 8.49, *p* = 0.011 • Duration of behaviours; RDE 23.44 M (16.67SD), SADE 9.04 M (11.58SD), *p* < 0.001 Significant difference between environments; F[1, 15] = 19.62, *p* < .001, ${Eta}^{2}$= 0.57 • Cooperation: SADE 3.31 and RDE 1.94. Significant effect; F = 33, *p* < 0.01 **EDA** • Tonic EDA before: RDE 428.88 M (444.54SD), SADE 1167.88 M (1038.16SD), *p* < 0.05 Significant difference between environments; F[1, 15] = 7.49, *p* < 0 .05, ${Eta}^{2}$= 0.33) • Phasic EDA during: RDE 446.06 M (455.90SD), SADE 1230.81 M (800.22SD), *p* < 0.01 Significant difference between environments; F[1, 15] = 15.34, *p* < .01, ${Eta}^{2}$ = 0.51 • Arousal**:** mean value of 413 kohms was found for the SADE, compared to 285 for the RDE. The paired *t* test was applied, and no significant difference (*p* = 0.32) • Relaxation: For the degree of relaxation, a mean value of 2,014 kohms was found for the SADE and 763 for the RDE. According to the *t* test, this difference was significant (*p* = 0.004)	• No significant effect of number of anxious behaviours • Significant positive differences on behaviour duration in SADE • Magnitude of behaviours significantly reduced in SADE • Cooperation significant increase in SADE • More relaxed in the SADE environment vs the RDE, but no significant difference was found in degree of arousal	“…to modulate conditions of physical environment to optimize participation, it may be necessary to find some means of individualizing sensory inputs” SADE could be an important substitute for pharmacological approaches “Importance of considering the sensory-adapted environment as a preferable dental environment for this population”. “1…. Innovative, non-invasive environmental approach to inducing relaxation and reducing anxiety in a dental clinic. 2. SADE requires no psychotherapeutic experience for dental personnel nor pharmacological sedation. 3. Simple to apply and has no negative side effects. 4. Model has potential to significantly provide oral and general health care for all children.”	SADE has positive calming effect that reduces behaviours and anxiety through the reduction of aversive stimuli. This successfully increases their engagement in dental procedures SADE produced positive effects on magnitude of behaviours, cooperation. SADE had no significant impact on arousal but found significant effect with relaxation scores and EDA	1. Could not accommodate observer blindness due to visible physical environment. However, EDA data enhances validity 2. Sample unable to determine effect of age, gender, and mental retardation level 3. Limited validity of NDBC
Cermak et al., 2015 [68]	**EDA:** NS-SCRs totalled for each participant and converted to fluctuations/min. Ratings counted if amplitude > / = to 0.05 **Effect sizes:** Cohen’s d for dental environment (RDE vs. SADE) within each group effect The randomized order of visits included as a priori covariate	**Behavioural distress (M +—SD, Effect size [Cohen’s d])** • CDBRS: RDE 47.3 ± 8.6 SADE 44.9 ± 11.8, **0.23** • A & C Scale: RDE 2.1 ± 1.6 SADE 1.8 ± 2.0, **0.13** • Frankl Scale: RDE 2.5 ± 1.0 SADE 2.7 ± 1.2, **0.21** **Physiological stress (EDA)** SCL • Exam + prophy + fluoride RDE 5.6 ± 3.9 SADE 3.6 ± 2.7, **0.65** • Exam + prophy RDE 5.6 ± 3.8, SADE 3.6 ± 2.5, **0.62** NSSCR Effect size = 4.6 • Exam + prophy + fluoride RDE 5.8 ± 3.3 SADE 4.6 ± 5.3**, 0.27** • Exam + prophy RDE 6.1 ± 3.5 SADE 4.4 ± 3.8, **0.46**	**Behavioural distress** • Environment effects on behavioural measures were not statistically significant but had small effects (d’s < 0.3) • CDBRS: Small effect of 0.23 • A & C Scale: negligible effect • Frankl Scale: Small effect between environments (*d* = 0.21) **Physiological stress (EDA)** SCL & NSSCR • Moderate to large effect (d’s = 0.27–0.65) • Statistically significant effect of environment for SCL exam + prophylaxis + fluoride and exam + prophylaxis (p’s = 0.01), and NS-SCR exam + prophylaxis (p = 0.05)	“SADE demonstrates utility and positive treatment effect sizes. SADE potential to improve dental care for ASD with dental anxiety and sensory processing difficulties.”	SADE produced small positive effects on behaviour. SADE demonstrated large impact on physiological stress, pain, and comfort. Overall, SADE demonstrates utility and positive effects in improving dental care for IDD population	1. Feasibility and pilot study and therefore not powered to detect differences 2. Lower functioning ASD sample 3. Consecutive sample with uneven gender distribution 4. 50% of ASD children were unable to complete self-report measures 5. Some outcome measures were not blinded to intervention 6. The social story prior may have led to unanticipated decreases in outcomes 7. Small sample size
Gowdham et al. 2021 [88]	Paired t-test was used for intragroup comparison and an unpaired t-test was used for intergroup comparison The level of statistical significance was set at a p value of < 0.05	**EDA:** *During dental examination* Group 1 Appointment 1—318.89 ± 204.998 Appointment 2—133.93 ± 77.074 Paired t = 3.095,* p* = 0.013 Unpaired t = 3.041, *p* = 0.007 Group 2 (without music) Appointment 1 – 144.93 ± 54.589 Appointment 2—329.76 ± 195.024 Paired t = − 3.827, *p* = 0.004 Unpaired t = − 2.953, *p* = 0.008 *During oral prophylaxis* Group 1 (with music) Appointment 1 – 369.43 ± 216.519 Appointment 2—149.73 ± 68.996 Paired t = 3.532, p = .006 Unpaired t = 3.076,* p* = 0.006 Group 2 (without music) Appointment 1 – 143.43 ± 84.244 Appointment 2—417.96 ± 194.142 Paired t = − 6.194, p = .000 Unpaired t = − 4.117, p value = 0.001 *During auditory operative stimuli* Group 1 Appointment 1 – 415.99 ± 243.427 Appointment 2—142.09 ± 69.700 Paired t = 4.305, p-.002 Unpaired t = 2.889, *p* = 0.009 Group 2 Appointment 1 – 163.23 ± 131.530 Appointment 2 – 468.63 ± 234.707 Paired t = − 6.003, p value = .000 Unpaired t = − 4.217, *p* = 0.000	A statistically significant increase in electrical resistance was observed during music distraction, which indicated an anxiety reduction when music distraction was employed	“The increased electrical skin resistance due to low anxiety proves the positive impact of music distraction in intellectually disabled children” Clinical significance “Music can be employed as a distraction technique to reduce anxiety in intellectually disabled children”	SADE produced positive effects on reducing anxiety in children with intellectual disability	1. Future studies invasive dental procedures and investigate duration of relaxation 2. Small sample size

*SADE* Sensory adapted environments, *RDE* Regular dental environment, *EDA* Electrodermal Activity NS-SCRs Non-specific skin conductance responses, *CDBRS* Children’s dental behaviour rating scale, *SCL* Skin conductance level, *NSSCR* Non-specific skin conductance responses, *EDA* Electrodermal Activity, *CDBRS* Children’s dental behaviour rating scale, *ACS* Anxiety cooperation scale FPS-R Faces Pain scale-revisits

### Maladaptive behaviours and cooperation

Cermak et al.[68] study found negligible to small effects (d’s = 0.13–23) for behavioural outcomes. Specifically, this study used the children’s dental behaviour rating scale. This scale rated children’s behaviour through video coding the presence or absence of distress behaviours (mouth movement, head movement and forehead movement), and severity of distress behaviours (cry or scream and verbal stall or delay). This study reported limited effect (*d* = 0.23) of children’s dental rating scale [68]. Cermak et al. [68] reported on the anxiety and cooperation scale, which is a dentist rating of behaviour during dental treatment. The results concluded negligible effect (*d* = 0.13) of SADE [68].

Another study by Kim et al. [60] reported on the effect of SADE on cooperation and behaviour using Frankl scale and a parent survey. This study found non statistically significant scores in paired analysis for clinicians (*p* = 0.07) and independent observer (*p* = 0.109) ratings using the Frankl scale. However, unpaired analysis demonstrated significant scores that accounted for large loss to follow-up for the clinicians (*p* = 0.037) and independent observer (*p* = 0.018) ratings. The parent questionnaire found that a large amount (92%—82%) of parents agreed/strongly agreed SADE improved cooperation, decrease dental anxiety and would prefer over RDE [60].

Shapiro et al. [86, 87] used the negative dental behaviour checklist to explore the impact of SADE on behaviours.

The results reported significant positive differences on the duration of accumulative anxious behaviours (*p* < 0.001) and magnitude (*p* = 0.011) of behaviours (whimpering as opposed to screaming) and between SADE and regular dental environments for EDA measures’.

However, there was no significant effect on the number of anxious behaviours (*p* = 0.19). Anxiety and cooperation scale was completed by the hygienist and the children showed significantly improved cooperation during treatment in SADE when compared with a regular dental environment (*p* < 0.01).

### Psychophysiological responses

The included studies reported varied psychophysiological responses of SADE. Kim et al. [60] used a pulse oximeter to measure heart rate and oxygen saturation and found no change. Cermak et al. [68] used multiple measure to access psychophysiological responses in SADE compared to RDE. Stress and anxiety levels were accessed via EDA measures of tonic skin conductance (SCL) and non-specific skin conductance responses (NS-SCRs). Both EDA measures via ANOCOVA models were small to moderate effect and found statistically significant difference between environments (d’s = 0.27–0.65;). Shapiro et al.[86, 87] compared tonic and phasic EDA measures. The results indicated significant differences of tonic (*p* < 0.05) and phasic (*p* < 0.01) EDA between environments. Shapiro et al.[86, 87] also analysed EDA to determine relaxation and arousal in each environment. This study found that children were significantly more relaxed in SADE than RDE correspondingly 2,014komns and 763komns (*p* = 0.004) and non-significant difference (*p* = 0.32) was found in degree of arousal with 413kohns and 285kohmns. Gowdham et al. [88] found a statistically significant increase in electrical resistance when music distraction is implemented in all groups with p-value ranging from 0.001- 0.009, providing strong evidence to support SADE in reducing dental anxiety.

### Meta-analyses

Meta-analyses were performed to determine the changes in EDA and behaviour between different interventions, SADE and RDE. Two studies reported data on these and were pooled to be included in the meta-analyses [68, 86]. The I^2^ statistic for each analysis demonstrated sufficient homogeneity to combine the studies. A random effects model was applied to account for heterogeneity. A sensitivity analysis for study quality was not possible due to the low number of included studies. The statistical results of the meta-analysis are presented in Additional file 8.

**Fig. 2:**
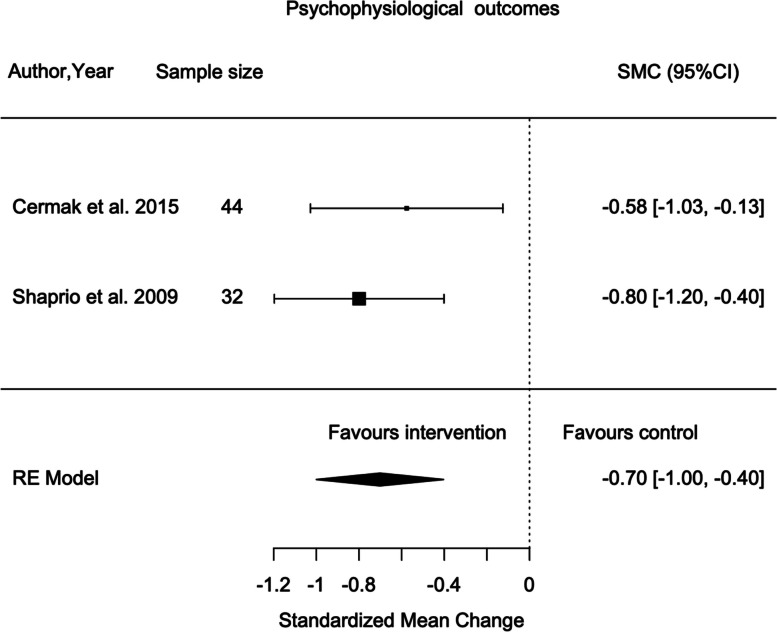
Meta-analysis for the effect of SADE vs. RDE on psychophysiological outcomes (EDA) during dental procedures

Data from two cross-over randomised trials (*n* = 38) were pooled to determine the effects of SADE on the changes of EDA, specifically phasic and non-specific EDA [68, 86]. The included studies showed that the effect of SADE was greater than RDE in decreasing anxiety specifically the corresponding psychophysiological responses. A statistically significant difference was found favouring SADE vs. RDE (Standardized mean change (SMC)-0.66; 95% CI -1.01 to -0.30; *p* < 0.001) (Fig. 2) [68, 86]. There was no evidence of heterogeneity (I2 = 0%; *p* = 0.468).

**Fig. 3:**
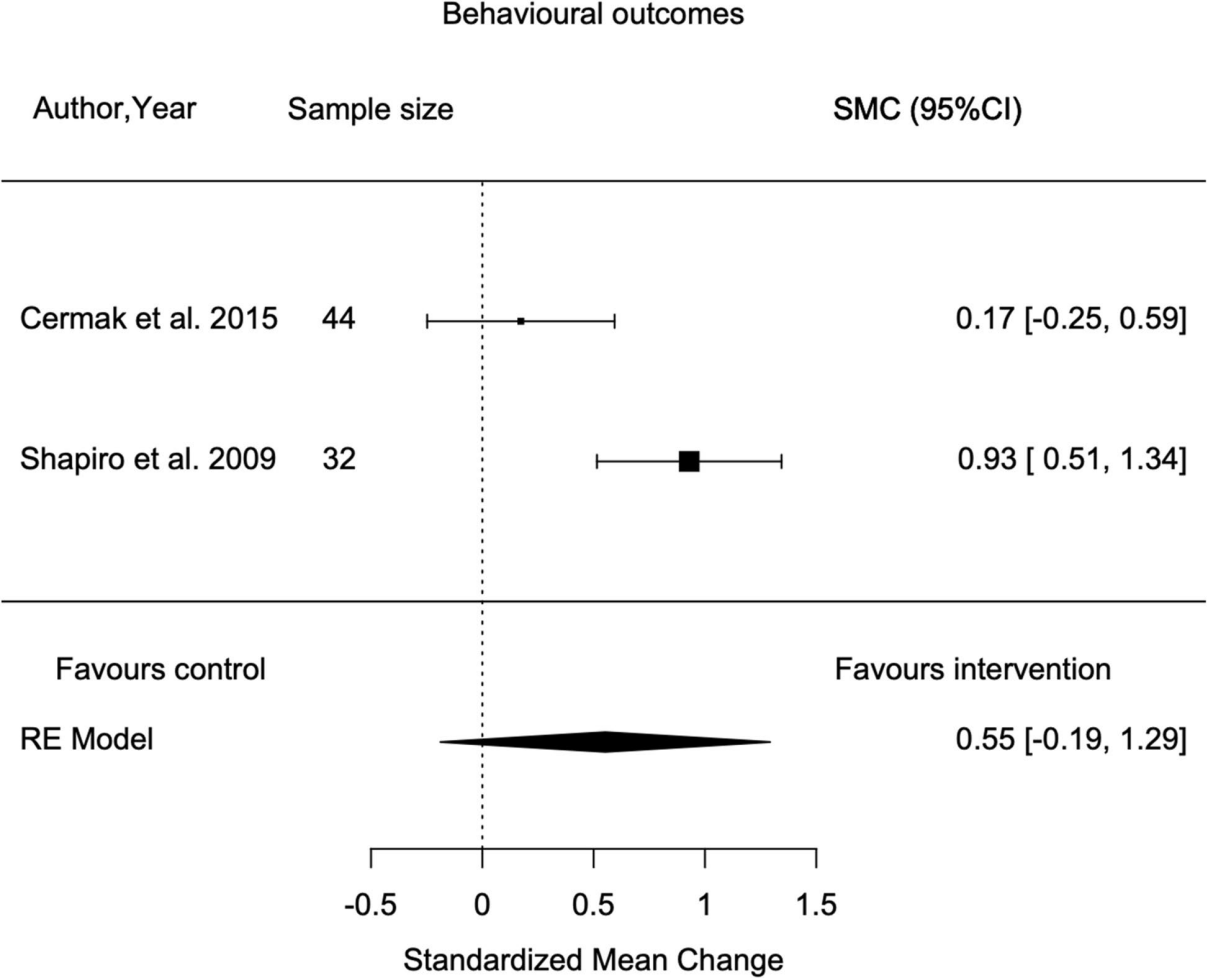
Meta-analysis for the effect of SADE vs. RDE on behaviour during dental procedures

Two cross-over randomised trials (*n* = 76) were pooled to determine the effects of SADE on the changes in behaviours using Frankl score and a behavioural checklist both rated by the dentist [68, 86]. Despite an overall tendency to favour the intervention group, no statistically significant difference was found between intervention and controls (SMC = 0.51; 95% CI -0.20 to 1.21; *p* = 0.161) (Fig. 3) [68, 86]. There was evidence of heterogeneity between the articles (I^2^ = 75%; *p* = 0.047).

### Risk of bias in studies

**Fig. 4:**
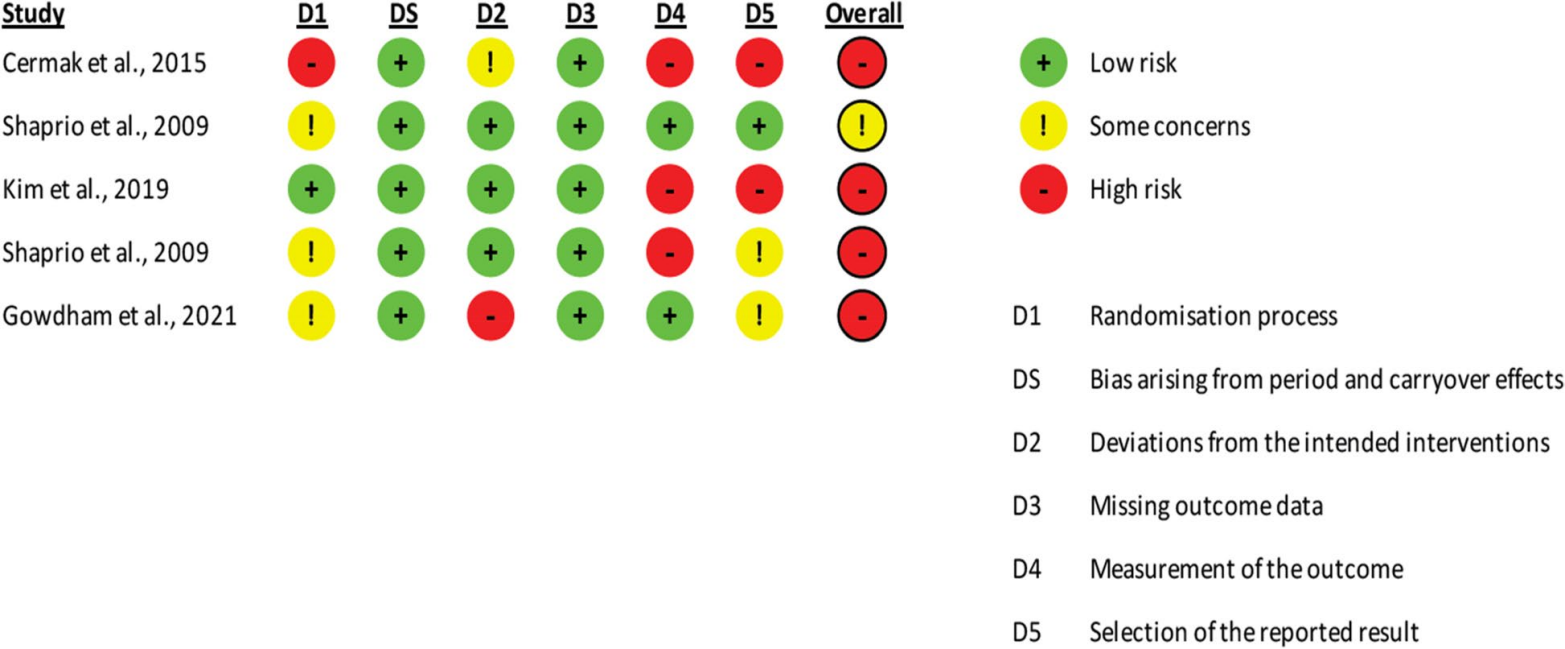
Risk of Bias traffic light figure of the included studies using the Cochrane ROB-2 for cross-over trials

**Fig. 5:**
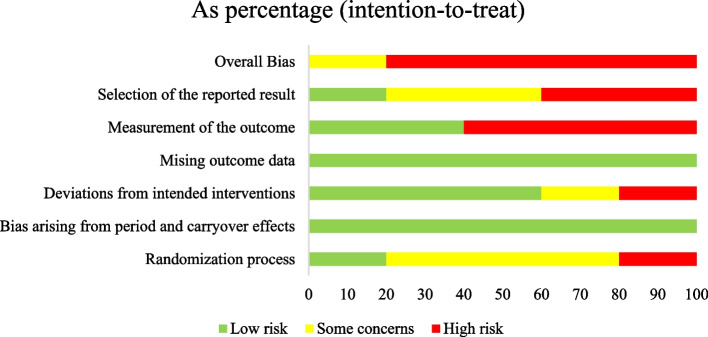
Risk of Bias summary of the included studies using the Cochrane ROB-2 for cross-over trials

The methodological quality of each study was assessed using the Cochrane ROB-2 for cross-over trials [78]. The results of the ROB assessment can be found tabulated in Additional file 7 and presented in Figs. 4 and 5. This tool revealed that the included studies in our systematic review varied in quality of methodology across the domains. One study had overall some concerns [74] and the four other studies were of high risk [60, 68, 87, 88]. Shaprio et al. [86, 87] reports used the same methodology with different outcome measures, therefore on basis of ROB each study was analysed separately. All studies were experimental randomised cross-over trials with small sample sizes. These studies showed limitations with respect to randomisation of participants, allocation concealment, blinding of participants, outcome assessors, intention to treat analysis, statistical power analysis and trial design [60, 68, 86–88]. No studies were excluded based on the risk of bias assessment.

### Selection bias – random sequence generation and allocation concealment

All included studies provided insufficient information about the randomisation process to determine the extent to which this may have affected the bias of these studies. Allocation concealment was not clear in most studies [68, 86–88] except in Kim et al. [60]. Baseline differences between groups were unclear due to differences between non-IDD population and people with IDD reported rather than individual group comparisons. However, Kim et al. [60] reported similar characteristics indicating randomisation was sufficient. Overall the studies were varied regarding selection bias; Cermak et al. [68] high, Shapiro et al. [86, 87] and Gowdham et al. [88] some concerns and Kim et al. [60] low risk.

### Carryover bias—bias arising from period and carryover effects

All studies reported carryover bias between treatment effects to be not significant [60, 68, 86–88]. Studies reported sufficient time (1–4 months) in-between interventions which is consistent elsewhere in the literature for usual time between oral procedures [60, 68, 86–88]. Therefore, all studies had a ‘low’ carry-over bias.

### Performance and detection bias – blinding of participants, assessors, and outcome assessment

Blinding of participants and assessors was not possible in any of the included studies due to the identifiable visible aspect of the intervention via environment/sensory modifications [60, 68, 86–88]. Furthermore, blinding of parent- and self-reported outcome measures was not feasible in all studies which increased the risk of detection bias [60, 68, 86–88]. Self-reported outcomes are inappropriate for people with IDD due to low IQ and expressive language. This influences detection bias, specifically in Cermak et al. [68] as half of the sample was unable to complete the questionnaires in the study. Therefore, a high risk of performance bias was considered in all studies as dentists that performed the procedure completed the outcome measures, impacting internal validity [60, 68, 86–88]. Multiple studies used external coders for behavioural outcome measures by using video recording that increased the validity and reliability of results due to low detection bias [60, 86, 87]. Outcome measures across studies included both appropriate and inappropriate tools. EDA was used in all studies [60, 68, 86–88]. However, EDA has limitations including inaccurate readings due to excessive movement, electrode placement and sensitivity. Although, it should be noted that Cermak et al. [68] employed a social story that may have improved compliance of electrodes, increasing accuracy of the readings. Hence, measurement of outcome domain was high for most studies [60, 68, 86] and low for Shapiro et al. [87] and Gowdham et al. [88].

### Attrition and reporting bias – incomplete outcome data and selective outcome reporting

Despite high attrition rate in Kim et al. [60], all data was combined in a unpaired analysis. The remainder of the included studies were deemed to have complete outcome data [68, 86–88]. Therefore, all studies were concluded ‘low’ risk for missing outcome data. The study by Gowdham et al. [88] and Shapiro et al.[86] ‘low’, Shapiro et al. [87] ‘some concerns’ and Cermak et al. [68] and Kim et al. [60] “high’ ROB of reported results.

## Discussion

The present review aimed to contribute to the emerging research for SADE targeted at children and young adults with IDD. Three multi-sensory dental environments were included in this review implementing various visual, tactile, and auditory adaptions [60, 68, 86, 87] and one single sensory using auditory input [88]. These studies demonstrated varying results regarding the effects of SADE on behaviour and psychophysiological responses of dental anxiety. However, due to the weakness of the studies, the accumulating evidence in the present studies only provides limited support to the assumption that the SADE can be used as an effective therapeutic tool. Dental anxiety has been conventionally managed using sedation methods to undertake simple dental cleaning [89]. Emerging non-pharmacological interventions have been investigated by an earlier systematic review by Phadraig et al. [47], although no strong evidence to support these approaches was found. Despite this, another recent review provided support of this approach to improve behaviour, anxiety, and pain by Goettems et al. [90]. Additionally, Ismail et al. [70] supports this reviews results that SADE is effective in reducing behaviour during dental treatment for children.

### Single sensory versus multi-sensory adaptation

Only one study included in this review explored single sensory adaptations using music distractibility in a dental setting. Gowdham et al. [88] found music to be effective in reducing anxiety levels. Evidence has observed a decrease in human emotional and physiological responses with music, indicating the potential to obviate the need for pharmacotherapy [91]. Similar previous reviews support this trend [92–94]. The systematic review by Bradt and colleagues [93] found a statistically significant anxiety reduction (*p* < 0.001) for preoperative music for children. Another meta-analyses by Kuhlmann et al. [92] reported statistically significant decrease in anxiety for adults (Mean difference (MD) –0·69, 95% CI–0·88 to –0·50; *p* < 0.001). Klassen et al. [94] found that music is an effective adjunctive therapeutic tool to reduce anxiety during medical and dental procedures. Therefore, an abundance of literature supports that music can be considered an adjunctive therapy in clinical situations to reduce anxiety. This review found no evidence to support reduction in maladaptive behaviours using single sensory based intervention during dental procedures as the included study failed to report the findings to address this outcome. Due to limited number of studies documenting single sensory approach, it is difficult to compare results with multi-sensory to examine the effectiveness. Although, majority of the studies support the notion that sensory adaptions are effective strategy to reduce anxiety for children and young adults with IDD [60, 68, 86–88]. Music was the only single sensory adaption included in this review [88]. Other single sensory techniques such as audio visual [95] television watching [96], use of virtual reality [97–99], deep pressure via papoose boards [57], noise attenuating headphones [100], and animals [101] are effective strategies in distracting the child’s attention from stress provoking situations. Although these studies were not included in this review as they did not match the inclusion criteria, further studies need to be conducted to confirm whether single or multi-sensory environments are more effective to guide dentists in addressing the needs of people with IDD.

### Maladaptive behaviours and cooperation

The results of this review revealed insufficient evidence supporting sensory adaptions effectiveness in reducing challenging or stereotypic self-stimulating behaviour in a dental setting [60, 68, 86, 87]. Frankl scale was used to analyse behaviour and cooperation in two studies [60, 68] and both reported a small positive effect of SADE on behaviour. The limited significance of the results could be due to the poor sensitivity of the 4-point scale to detect change due to classification not providing definite items for observation [102]. Shapiro et al. [86, 87] found no effect on the number of anxious behaviours but reduced magnitude and increased relaxation during SADE. Thus, the outcomes of the included studies demonstrated a small effect of SADE on behaviour [60, 68, 86, 87]. A large volume of evidence supports Snoezelen as an approach for people with IDD to reduce maladaptive behaviours in multiple settings and diagnoses including brain injury, dementia, schools, and hospitals [61–66, 103]. The sensory processing difficulties with modulation or discrimination have been linked to increased maladaptive behaviours [38–40]. These maladaptive behaviours are due to sensory defensiveness as a result from extreme avoidance or behavioural overreaction to certain sensory experiences [104]. Included studies in this review support that modifying sensory stimuli in a dental setting improve the dental experience for people with disabilities, reducing the magnitude of behaviours [68, 87]. Likewise, studies have documented trends of decreased disruptive behaviour in Snoezelen interventions for people with intellectual and developmental disabilities [66].

The meta-analyses involving two studies uncovered inconsistent results of the impact of SADE on behaviour [68, 86]. It is, however, imperative to recognise that the included studies used varying outcome measures contributing to high heterogeneity. Hence, these results should be interpreted with caution due to the inconsistency of results that cross the line of no effect. Contrastingly, a meta-analyses on the effectiveness of Snoezelen in populations with IDD found significant and large effect size (0.63 to 2.63) in adaptive behaviours, although not significant due to small sample and heterogeneity [105]. It should be noted that Kim [60] used the Frankl score although it was excluded from the meta-analysis as only the mean was reported and no standard deviation. The authors made three attempts to contact the author but no reply was received. Therefore, the study was not included in the meta-analysis. In all, considering this review and previous research [60, 68, 86, 87], there is no recent evidence of well-designed studies in support of SADE to reduce maladaptive behaviours, only small positive effects are established that may improve the administration of oral care.

### Psychophysiological responses

Dental anxiety provokes physical symptoms detailed as sweating, decreased gastrointestinal motility and cutaneous vasoconstriction [106]. It is typical that individuals with increased anxiety levels may experience elevated psychophysiological responses [107]. This is consistent with the three included studies in this review that demonstrated significant decrease in psychophysiological responses using Snoezelen dental environments [68, 86–88]. However, one study reported no change [60]. Although this result must be evaluated with caution due to inaccurate EDA readings due to excessive movement of participants [60]. The meta-analyses of the two included studies found a statistically significant reduction in psychophysiological responses of anxiety in SADE [68, 86]. These findings are consistent with other studies analysing the effect of sensory adaptions on reducing anxiety in various settings and diagnoses including dementia [108–110] and brain injury [111–113]. More importantly, SADE has shown improvements in populations with IDD within literature [68, 86–88]. Specifically, a study suggests that the activation of parasympathetic and sympathetic nervous systems plays a critical role in autonomic nervous system modulation using deep touch pressure via weighted blanket that reduces dental anxiety [114]. Thus, sound evidence supports sensory adaptions capacity to reduce anxiety present in stress provoking situations, particularly the dental environment [68, 86–88].

### Strengths and limitations

The strengths of this review are that its findings are based on only RCT studies to understand whether SADE is effective in reducing maladaptive behaviours and psychophysiological outcomes of dental anxiety for people with IDD. We performed an exhaustive literature search (six electronic databases and citation searching) without language restriction to ensure we captured all relevant evidence on the topic of interest, thereby reducing the chances of selection bias. ROB-2 for cross-over studies is a widely recognised tool that was used to assess the methodological quality and ROB of the included studies. This review also followed PRISMA thoroughly therefore others can replicate this review and adds to the overall quality of this review.

The findings of this systematic review and meta-analyses should be considered on reflection of several limitations. The review is limited by the relatively small amount of studies that met the eligibility criteria. The sample sizes of the included studies were small ranging from 16–22 participants [60, 68, 86–88], thereby reducing the power of the study and increasing margin of error in the results. The results of this review may not be generalised to individuals with IDD due to large heterogeneity in ages, diagnosis, and severity as well as limited representation of females and ethnic minorities. Future studies should allow for sub-group analysis. All participants in the included studies had below average expressive communication, therefore the studies captured lower functioning or moderate-severe IDD [60, 68, 86–88]. The included studies were of a questionable methodological quality weakening results of this review due to inadequate blinding, allocation concealment and report of results. In addition, we were not able to formally assess publication bias. Nevertheless, we are confident that these methodological limitations would not change the overall conclusions of this review.

### Future research

The results give some initial support to the assumption that the SADE has value as a therapeutic dental approach; yet further rigorous research would enable the confidence and generalisability of this assumption. The Short Sensory Profile 2 was utilised to identify SI difficulties across two included studies in this review [60, 68]. However, no study utilised these profiles to customise the sensory adaptations to accommodate sensory differences to enable regulation [115] and greater cooperative in procedures. Future studies should adopt this approach to support arousal and reduce sensory reactivity within the dental environment. Majority of the included studies failed to gain clients’, parents’, and dentists’ perspectives using qualitative methodology [60, 86–88]. Future studies should adopt qualitative design to gain valuable in-depth understanding of experiences using SADE. All included studies were limited to oral examination, prophylaxis [60, 68, 86–88] and fluoride application [60]. It is recommended that future research explore other dental procedures including tooth extractions, tooth fillings or orthodontic treatment. It is recommended that other aspects of dental setting are altered to target certain sensory patterns including calming scents, noise cancelling headphones or silencing dental tools. To increase generalisability to the broader population with IDD, a sample with high or mild functioning need to be included in future studies. This will also increase the appropriateness of child-reported measures. The current understanding of SADE generalisability is limited to India [88], America [60, 68] and Israel [86, 87].Therefore, high quality research in additional countries are needed. All included studies had small sample sizes (16–22 participants) [60, 68, 86–88]. Future research should investigate SADE using high quality studies with larger sample sizes. No studies were located to address SADE in the context of the waiting room. Further studies should address whether pre-procedural sensory adaptions can reduce dental anxiety. A case control study of typical developing population found no significant difference in dental anxiety in sensory adapted waiting room, but significantly higher dental anxiety for visit purpose and waiting time [116]. Despite this limited evidence children included had low overall anxiety, therefore impacting the result [116]. Thus, it can be expected that individuals with IDD would benefit from sensory adaptions in the waiting room and is recommended for future studies to address this research gap.

### Practice implications

This review provides evidence that SADE can be effective at reducing behaviours associated with dental anxiety. However, current evidence is limited regarding the benefits of reducing the psychophysiological and behavioural responses of dental anxiety. Therefore, generalising to practice should be done with caution due to questionable risk of bias highlighted in the included studies. The main recommendation for practice is the need for interprofessional education and collaborative practice between occupational therapists (OT) and dental practitioners. This has been associated with greater quality of care, patient safety and health outcomes [117, 118]. In addition it promotes greater understanding of scopes of practice and innovative clinical approaches [119]. Evidence reports dental practitioners’ inadequacies in their knowledge, training, and exposure to treating children with IDD [120–123]. It is clear from the conclusion made from this review that there is potential for OT collaboration in a dental setting. OTs have unique specialised training in task analysis, sensory adaptations, and ecological models of practice that could be used to capture oral health barriers specifically in the dental procedure or waiting room [124, 125]. Therefore, collaboration with OTs and dentists is highly recommended to increase competencies of dentists to address individuals with IDD needs.

## Conclusions

Although this review included only a small number of studies, there is some evidence that SADE could be a promising intervention for reducing dental anxiety among children and young adults with IDD. The meta-analyses showed SADE can be effective in reducing psychophysiological outcomes, however uncovered limited and inconsistent effects on behaviour. Based on the narrative synthesis, adapting visual, tactile, and auditory aspects of the dental environment demonstrates small positive effects on dental anxiety. Future studies need to be incorporate the uniqueness of sensory profiles and individualised adaptions accordingly. The apparent SI difficulties experienced by this population and positive benefits of SADE highlights the clear scope for OT in a dental setting to address their complex needs. Future researchers should be encouraged to continue this line of research, to further support SADE in clinical dental practice.

### Supplementary Information

**Additional file 1. **PRISMA checklist.

**Additional file 2. **Previous systematic review summary.

**Additional file 3. **PICO framework.

**Additional file 4. **Search strategy.

**Additional file 5. **Reasons for exclusion of studies.

**Additional file 6. **Quality assessment of included studies.

**Additional file 7. **Intervention criteria.

**Additional file 8. **Meta-analysis results for the effect of SADE vs. RDE on EDA and behavioural outcomes during dental procedures.

## Data Availability

All data generated or analysed during this study are included in this published article [and its supplementary information files].

## Abbreviations

SADE: Sensory Adapted Dental Environment
IDD: Intellectual and Developmental Disabilities
RDE: Regular Dental Environment
OT: Occupational Therapy
RCT: Randomised Control Trials
SI: Sensory Integration
RDE: Regular Dental Environment
PRISMA: Preferred Reporting Items for Systematic Reviews and Meta-Analyses
PROSPERO: International Prospective Register of Systematic Reviews
ICF: International Classification of Functioning
MESH: Medical Subject Headings
ADHD: Attention Deficit Hyperactivity Disorder
EDA: Electrodermal Activity
MD: Mean Difference
SMC: Standardised Mean Change
ASD: Autism Spectrum Disorder
PICOS: Population Intervention Comparator Outcome and Study Design
CI: Confidence Intervals
ROB: Risk of Bias

## References

[1] Schalock RL. Intellectual disability. Cross-Cultural Psychology: Contemporary Themes and Perspectives. 2010, pp. 312.

[2] American Psychiatric Association. Diagnostic and statistical manual of mental disorders : DSM-5. Fifth edition ed. Washington, DC: Washington, DC: American Psychiatric Publishing; 2013.

[3] Anderson LL, Larson SA, MapelLentz S, Hall-Lande J (2019). A Systematic Review of U.S. Studies on the Prevalence of Intellectual or Developmental Disabilities Since 2000. Intellect Dev Disabil.

[4] Ansari H. Magnitude of Developmental Disabilities in India. Birth Defects in India. 2021, pp. 169–94.

[5] Madden R (2004). Estimates of prevalence of intellectual disability in Australia. J Intellect Dev Dis.

[6] Emerson E (2012). Deprivation, ethnicity and the prevalence of intellectual and developmental disabilities. J Epidemiology Community Health.

[7] Brennan DS, Spencer AJ, Roberts-Thomson KF (2019). Socioeconomic and psychosocial associations with oral health impact and general health. Community Dent Oral Epidemiol.

[8] Sischo L, Broder H (2011). Oral health-related quality of life: what, why, how, and future implications. J Dent Res.

[9] Song J-S,  Hyun H-K, Shin TJ, Kim Y-J (2018). Effects of dental treatment and systemic disease on oral health-related quality of life in Korean pediatric patients. BMC Oral Health.

[10] Ward L, Cooper S, Hughes-McCormack L, Macpherson L, Kinnear D. Oral health of adults with intellectual disabilities: a systematic review. J Intellect Disabil Res 2019;63(11):1359–78. 10.1111/jir.12632 31119825

[11] Anders PL, Davis EL (2010). Oral health of patients with intellectual disabilities: a systematic review. Spec Care Dentist.

[12] Allerton LA, Welch V, Emerson E (2011). Health inequalities experienced by children and young people with intellectual disabilities: A review of literature from the United Kingdom. J Intellect Disabil.

[13] Zhou N, Wong HM, Wen YF, McGrath C (2017). Oral health status of children and adolescents with intellectual disabilities: a systematic review and meta-analysis. Dev Med Child Neurol.

[14] Wilson NJ, Lin Z, Villarosa A, George A (2019). Oral health status and reported oral health problems in people with intellectual disability: A literature review. J Intellect Dev Disabil.

[15] Wilson NJ, Lin Z, Villarosa A, Lewis P, Philip P, Sumar B (2019). Countering the poor oral health of people with intellectual and developmental disability: a scoping literature review. BMC Public Health.

[16] Emerson E, Baines S, DuBois L, Welch V. Health inequalities and people with learning disabilities in the UK; 2010.: Improving Health and Lives: Learning Disabilities Observatory; 2011.

[17] Petrovic BB, Peric TO, Markovic DL, Bajkin BB, Petrovic D, Blagojevic DB (2016). Unmet oral health needs among persons with intellectual disability. Res Dev Disabil.

[18] Feldberg I (2014). Merrick J.

[19] da Rosa SV, Moysés SJ, Theis LC, Soares RC, Moysés ST, Werneck RI (2020). Barriers in access to dental services hindering the treatment of people with disabilities: a systematic review. Int J Dent.

[20] Nicolaidis C, Raymaker DM, Ashkenazy E, McDonald KE, Dern S, Baggs AE (2015). “Respect the way I need to communicate with you”: Healthcare experiences of adults on the autism spectrum. Autism.

[21] Thomas N, Blake S, Morris C, Moles DR (2018). Autism and primary care dentistry: parents’ experiences of taking children with autism or working diagnosis of autism for dental examinations. Int J Paediatr Dent.

[22] Du RY, Yiu CK, King NM (2019). Oral health behaviours of preschool children with autism spectrum disorders and their barriers to dental care. J Autism Dev Disord.

[23] Alshatrat SM, Al-Bakri IA, Al-Omari WM (2020). Dental service utilization and barriers to dental care for individuals with autism spectrum disorder in Jordan: a case-control study. Int J Dent.

[24] Byrappagari D, Jung Y, Chen K (2018). Oral health care for patients with developmental disabilities: a survey of Michigan general dentists. Spec Care Dentist.

[25] Leal AMA, Serra KG, Queiroz RCS, Araujo MAR, Maia Filho EM (2013). Fear and/or anxiety of children and parents associated with the dental environment. Eur J Paediatr Dent.

[26] Armfield JM (2010). Towards a better understanding of dental anxiety and fear: cognitions vs. experiences. Eur J Oral Sci.

[27] Assunção C, Losso E, Andreatini R, de Menezes JV (2013). The relationship between dental anxiety in children, adolescents and their parents at dental environment. J Indian Soc Pedod Prev Dent.

[28] Lee J, Chang J (2021). Oral health issues of young adults with severe intellectual and developmental disabilities and caregiver burdens: a qualitative study. BMC Oral Health.

[29] Chadwick D, Chapman M, Davies G (2018). Factors affecting access to daily oral and dental care among adults with intellectual disabilities. J Appl Res Intellect Disabil.

[30] Dahlander A, Soares F, Grindefjord M, Dahllöf G (2019). Factors associated with dental fear and anxiety in children aged 7 to 9 years. Dent J.

[31] Gaffar BO, Alagl AS, Al-Ansari AA (2014). The prevalence, causes, and relativity of dental anxiety in adult patients to irregular dental visits. Saudi Med J.

[32] Keleş S, Abacıgil F, Adana F, Yeşilfidan D, Okyay P (2018). The association between dental anxiety and oral health related quality of life among individuals with mild intellectual disability. Meandros med dental j.

[33] Grisolia BM, dos Santos APP, Dhyppolito IM, Buchanan H, Hill K, Oliveira BH (2021). Prevalence of dental anxiety in children and adolescents globally: A systematic review with meta-analyses. Int J Paediatr Dent.

[34] Fallea A,  Zuccarello R, Calì F (2016). Dental anxiety in patients with borderline intellectual functioning and patients with intellectual disabilities. BMC Oral Health.

[35] Ayres AJ (1964). Tactile functions. Their relation to hyperactive and perceptual motor behavior. Am J Occup Ther.

[36] Galiana-Simal A, Vela-Romero M, Romero-Vela VM, Oliver-Tercero N, García-Olmo V, Benito-Castellanos PJ (2020). Sensory processing disorder: Key points of a frequent alteration in neurodevelopmental disorders. Cogent Medicine.

[37] Dellapiazza F, Michelon C, Oreve M-J, Robel L, Schoenberger M, Chatel C (2019). The Impact of Atypical Sensory Processing on Adaptive Functioning and Maladaptive Behaviors in Autism Spectrum Disorder During Childhood: Results From the ELENA Cohort. J Autism Dev Disord.

[38] Cheung PPP, Siu AMH (2009). A comparison of patterns of sensory processing in children with and without developmental disabilities. Res Dev Disabil.

[39] Shimizu VT, Bueno OFA, Miranda MC (2014). Sensory processing abilities of children with ADHD. Braz J Phys Ther.

[40] Blanche EI, Reinoso G, Chang MC, Bodison S (2012). Proprioceptive Processing Difficulties Among Children With Autism Spectrum Disorders and Developmental Disabilities. Am J Occup Ther.

[41] Stein LI, Polido JC, Mailloux Z, Coleman GG, Cermak SA (2011). Oral care and sensory sensitivities in children with autism spectrum disorders. Spec Care Dentist.

[42] Allred KD, Byers JF, Sole ML (2010). The Effect of Music on Postoperative Pain and Anxiety. Pain Manag Nurs.

[43] Singh D, Samadi F, Jaiswal J, Tripathi AM (2014). Stress Reduction through Audio Distraction in Anxious Pediatric Dental Patients: An Adjunctive Clinical Study. Int J Clin Pediatr Dent.

[44] Cochrane Handbook for Systematic Reviews of Interventions version 6.3 (updated February 2022): Cochrane, 2022. . Available from: www.training.cochrane.org/handbook. .

[45] Olivieri JG, de España C, Encinas M, Ruiz X-F, Miró Q, Ortega-Martinez J (2021). Dental Anxiety, Fear, and Root Canal Treatment Monitoring of Heart Rate and Oxygen Saturation in Patients Treated during the Coronavirus Disease 2019 Pandemic: An Observational Clinical Study. J Endod.

[46] Hoehn-Saric R, McLeod DR (2000). Anxiety and arousal: physiological changes and their perception. J Affect Disord.

[47] Mac Giolla Phadraig C, Asimakopoulou K, Daly B, Nunn J (2020). Nonpharmacological techniques to support patients with intellectual developmental disorders to receive dental treatment: A systematic review of behavior change techniques. Spec Care Dentist.

[48] Chang J, Patton LL, Kim H-Y (2014). Impact of dental treatment under general anesthesia on the oral health-related quality of life of adolescents and adults with special needs. Eur J Oral Sci.

[49] Akpinar H (2019). Evaluation of general anesthesia and sedation during dental treatment in patients with special needs: A retrospective study. J Dent Anesth Pain Med.

[50] Fu D, Lopez-Silva C, Walsh LJ, Pradhan A (2021). Conscious sedation, general anaesthesia for patients with special needs. Int Dent J.

[51] Newton JT (2009). Restrictive behaviour management procedures with people with intellectual disabilities who require dental treatment. J Appl Res Intellect Disabil.

[52] Martins-Junior PA (2017). Dental treatment under general anaesthetic and children's oral health-related quality of life: Question: What is the impact of dental treatment under general anaesthesia on children's oral health-related quality of life?. Evid Based Dent.

[53] LeBel J, Nunno MA, Mohr WK, O'Halloran R (2012). Restraint and seclusion use in U.S. school settings: Recommendations from allied treatment disciplines. Am J Orthopsychiatry.

[54] Deshais MA, Fisher AB, Hausman NL, Kahng S (2015). Further investigation of a rapid restraint analysis. J APPL BEHAV ANAL.

[55] Kasemkhun P, Smutkeeree A, Jirarattanasopha V (2022). A retrospective comparison of dental treatment under general anesthesia versus non-pharmacological approach in patient with special health care needs. J Dent Sci.

[56] Chavis SE, Wu E, Munz SM (2021). Considerations for Protective Stabilization in Community General Dental Practice for Adult Patients with Special Healthcare Needs. Compend Contin Educ Dent.

[57] Chen H-Y, Yang H, Chi H-J, Chen H-M (2014). Physiologic and behavioral effects of papoose board on anxiety in dental patients with special needs. J Formos Med Assoc.

[58] Fowler Sa. Multisensory rooms and environments : controlled sensory experiences for people with profound and multiple disabilities. 1st American paperback. ed. Pagliano Pwof, ProQuest, Ebook C, editors. London Philadelphia: London Philadelphia : Jessica Kingsley Publishers; 2008.

[59] Potter CN, Wetzel JL, Learman KE (2019). Effect of sensory adaptations for routine dental care in individuals with intellectual and developmental disabilities: A preliminary study. Intellect Dev Disabil.

[60] Kim G, Carrico C, Ivey C, Wunsch PB (2019). Impact of sensory adapted dental environment on children with developmental disabilities. Spec Care Dentist.

[61] Cuvo AJ, May ME, Post TM (2001). Effects of living room, Snoezelen room, and outdoor activities on stereotypic behavior and engagement by adults with profound mental retardation. Res Dev Disabil.

[62] Kwok HWM, To YF, Sung HF (2003). The application of a multisensory Snoezelen room for people with learning disabilities-Hong Kong experience. Hong Kong Med J.

[63] McKee SA, Harris GT, Rice ME, Silk L (2007). Effects of a Snoezelen room on the behavior of three autistic clients. Res Dev Disabil.

[64] Novakovic N, Milovancevic MP, Dejanovic SD, Aleksic B (2019). Effects of Snoezelen—Multisensory environment on CARS scale in adolescents and adults with autism spectrum disorder. Res Dev Disabil.

[65] Carter M, Stephenson J (2011). The use of multi-densory environments in schools servicing children with severe disabilities. J Dev Phys Disabil.

[66] Fava L, Strauss K (2010). Multi-sensory rooms: Comparing effects of the Snoezelen and the Stimulus Preference environment on the behavior of adults with profound mental retardation. Res Dev Disabil.

[67] Shapiro M, Melmed RN, Sgan-Cohen HD, Eli I, Parush S (2007). Behavioural and physiological effect of dental environment sensory adaptation on children's dental anxiety. Eur J Oral Sci.

[68] Cermak SA, Stein Duker LI, Williams ME, Dawson ME, Lane CJ, Polido JC (2015). Sensory Adapted Dental Environments to Enhance Oral Care for Children with Autism Spectrum Disorders: A Randomized Controlled Pilot Study. J Autism Dev Disord.

[69] Bodison SC, Diane PL (2018). Specific sensory techniques and sensory environmental nodifications for children and youth With sensory integration difficulties: a systematic review. Am J Occup Ther.

[70] Ismail A, Tengku Azmi T, Malek W, Mallineni S (2021). The effect of multisensory-adapted dental environment on children's behavior toward dental treatment: A systematic review. J Indian Soc Pedod Prev Dent.

[71] Page MJ, McKenzie JE, Bossuyt PM, Boutron I, Hoffmann TC, Mulrow CD (2021). The PRISMA 2020 statement: an updated guideline for reporting systematic reviews. BMJ.

[72] Reynolds K, Chandio N, Chimoriya R, Arora A. The effectiveness of sensory adaptive dental environments to reduce dental anxiety and corresponding negative behaviours and psychophysiology responses in children and young adults with intellectual and developmental disabilities: a systematic review protocol. PROSPERO: International prospective register of systematic reviews 2022. 10.3390/ijerph192113758 PMC9654101 36360634

[73] Reynolds K, Chandio N, Chimoriya R, Arora A (2022). The Effectiveness of Sensory Adaptive Dental Environments to Reduce Corresponding Negative Behaviours and Psychophysiology Responses in Children and Young People with Intellectual and Developmental Disabilities: A Protocol of a Systematic Review and Meta-Analysis. Int J Environ Res Public Health.

[74] Costantino G, Montano N, Casazza G (2015). When should we change our clinical practice based on the results of a clinical study? Searching for evidence: PICOS and PubMed. Intern Emerg Med.

[75] Organization WH. Towards a common language for functioning, disability, and health: ICF. The international classification of functioning, disability and health. 2002.

[76] Faulks D, Norderyd J, Molina G, Macgiolla Phadraig C, Scagnet G, Eschevins C (2013). Using the International Classification of Functioning, Disability and Health (ICF) to describe children referred to special care or paediatric dental services. PLoS ONE.

[77] The EndNote Team. EndNote. EndNote X9 ed. Philadelphia, PA: Clarivate; 2013.

[78] Higgins JP, Altman DG, Gøtzsche PC, Jüni P, Moher D, Oxman AD (2011). The Cochrane Collaboration's tool for assessing risk of bias in randomised trials. BMJ.

[79] Hoffmann T, Bennett S, Del Mar CB (2017). Evidence-based practice across the health professions-E-pub.

[80] Polgar S, Thomas SA. Introduction to research in the health sciences Elsevier Health Sciences; 2011.

[81] R Core Team (2022). R: A Language and Environment for Statistical Computing.

[82] Tufanaru C, Munn Z, Stephenson M, Aromataris E (2015). Fixed or random effects meta-analysis? Common methodological issues in systematic reviews of effectiveness. Int J Evid Based Healthc.

[83] Higgins JP, Thomas J, Chandler J, Cumpston M, Li T, Page MJ, et al. Cochrane handbook for systematic reviews of interventions: John Wiley & Sons; 2019. 10.1002/14651858.ED000142 PMC10284251 31643080

[84] Higgins JP, Thompson SG, Deeks JJ, Altman DG (2003). Measuring inconsistency in meta-analyses. BMJ.

[85] Ades A, Lu G, Higgins J (2005). The interpretation of random-effects meta-analysis in decision models. Med Decis Making.

[86] Shapiro M, Melmed RN, Sgan-Cohen HD, Parush S (2009). Effect of sensory adaptation on anxiety of children with developmental disabilities: a new approach. Pediatr Dent.

[87] Shapiro M, Sgan-Cohen HD, Parush S, Melmed RN (2009). Influence of adapted environment on the anxiety of medically treated children with developmental disability. J Pediatr.

[88] Gowdham G, Shetty AA, Hegde AM, Suresh LR (2021). Impact of Music Distraction on Dental Anxiety in Children Having Intellectual Disability. Int J Clin Pediatr Dent.

[89] Wang Y-C, Lin I-H, Huang C-H, Fan S-Z (2012). Dental anesthesia for patients with special needs. Acta Anaesthesiol Taiwan.

[90] Goettems ML, Zborowski EJ, Costa FD, Costa VP, Torriani DD (2017). Nonpharmacologic Intervention on the Prevention of Pain and Anxiety During Pediatric Dental Care: A Systematic Review. Acad Pediatr.

[91] Crowe BJ (2004). Music and soulmaking: Toward a new theory of music therapy. J Music Ther.

[92] Kühlmann AYR, de Rooij A, Kroese LF, van Dijk M, Hunink MGM, Jeekel J (2018). Meta-analysis evaluating music interventions for anxiety and pain in surgery. Br J Surg.

[93] Bradt J, Dileo C, Shim M. Music interventions for preoperative anxiety. Cochrane Database Syst Rev. 2013(6). 10.1002/14651858.CD006908.pub2 PMC9758540 23740695

[94] Klassen JA, Liang Y, Tjosvold L, Klassen TP, Hartling L (2008). Music for pain and anxiety in children undergoing medical procedures: a systematic review of randomized controlled trials. Ambul Pediatr.

[95] Bagattoni S, D'Alessandro G, Sadotti A, Alkhamis N, Piana G (2018). Effects of audiovisual distraction in children with special healthcare needs during dental restorations: a randomized crossover clinical trial. Int J Paediatr Dent.

[96] Ghadimi S, Estaki Z, Rahbar P, Shamshiri A (2018). Effect of visual distraction on children’s anxiety during dental treatment: a crossover randomized clinical trial. Eur Arch Paediatr Dent.

[97] López-Valverde N, Muriel Fernández J, López-Valverde A, Valero Juan LF, Ramírez JM, Flores Fraile J (2020). Use of virtual reality for the management of anxiety and pain in dental treatments: Systematic review and meta-analysis. J Clin Med.

[98] Lahti S, Suominen A, Freeman R, Lähteenoja T, Humphris G (2020). Virtual reality relaxation to decrease dental anxiety: Immediate effect randomized clinical trial. JDR Clin Trans Res.

[99] Suresh LR, George C (2019). Virtual reality distraction on dental anxiety and behavior in children with autism spectrum disorder. J Int Dent Medical Res.

[100] Pfeiffer B, Stein Duker L, Murphy A, Shui C. Effectiveness of noise-attenuating headphones on physiological responses for children with autism spectrum disorders. Front Integr Neurosci. 2019;13. 10.3389/fnint.2019.00065 PMC6863142 31798424

[101] Thakkar T, Naik S, Dixit U (2021). Assessment of dental anxiety in children between 5 and 10 years of age in the presence of a therapy dog: A randomized controlled clinical study. Eur Arch Paediatr Dent.

[102] Narayan V, Samuel S (2019). Appropriateness of various behavior rating scales used in pediatric dentistry: A Review. J Glob Oral Health.

[103] Bergstrom VN, O’Brien-Langer A, Marsh R (2019). Supporting children with fetal alcohol spectrum disorder: Potential applications of a Snoezelen multisensory room. Phys Occup Ther Pediatr.

[104] Lane SJ, Mailloux Z, Schoen S, Bundy A, May-Benson TA, Parham LD (2019). Neural foundations of Ayres Sensory Integration®. Brain Sci.

[105] Lotan M, Gold C (2009). Meta-analysis of the effectiveness of individual intervention in the controlled multisensory environment (Snoezelen®) for individuals with intellectual disability. J Intellect Dev Disabil.

[106] Morgan AG, Rodd HD, Porritt JM, Baker SR, Creswell C, Newton T (2017). Children's experiences of dental anxiety. Int J Paediatr Dent.

[107] Steimer T (2002). The biology of fear- and anxiety-related behaviors. Dialogues Clin Neurosci.

[108] Sacks AL (2005). Effects of Snoezelen behavior therapy on increasing independence in activities of daily living of elders with dementia on a short term geriatric psychiatric unit. Int J Psychiatry Med.

[109] Pinto JO, Dores AR, Geraldo A, Peixoto B, Barbosa F (2020). Sensory stimulation programs in dementia: A systematic review of methods and effectiveness. Expert Rev Neurother.

[110] Sánchez A, Maseda A, Marante-Moar MP, De Labra C, Lorenzo-López L, Millán-Calenti JC (2016). Comparing the effects of multisensory stimulation and individualized music sessions on elderly people with severe dementia: a randomized controlled trial. J Alzheimers Dis.

[111] Hotz GA, Castelblanco A, Lara IM, Weiss AD, Duncan R, Kuluz JW (2006). Snoezelen: A controlled multi-sensory stimulation therapy for children recovering from severe brain injury. Brain Inj.

[112] Gómez C, Poza J, Gutiérrez MT, Prada E, Mendoza N, Hornero R (2016). Characterization of EEG patterns in brain-injured subjects and controls after a Snoezelen® intervention. Comput Methods Programs Biomed.

[113] Koller D, McPherson AC, Lockwood I, Blain-Moraes S, Nolan J (2018). The impact of Snoezelen in pediatric complex continuing care: A pilot study. J Pediatr Rehabil Med.

[114] Chen H-Y, Yang H, Chi H-J, Chen H-M (2013). Physiological effects of deep touch pressure on anxiety alleviation: The weighted blanket approach. J Med Biol Eng.

[115] Dunn W (2007). Supporting children to participate successfully in everyday life by using sensory processing knowledge. Infants Young Child.

[116] Fux-Noy A, Zohar M, Herzog K, Shmueli A, Halperson E, Moskovitz M (2019). The effect of the waiting room’s environment on level of anxiety experienced by children prior to dental treatment: a case control study. BMC Oral Health.

[117] Gupta B, Nanda A, Jain V, Verma M (2017). Interprofessional education: A reform plan for collaborative. Contemp Clin Dent.

[118] Kanji Z, Lin D, Krekoski C (2017). Interprofessional education and collaborative practice. Can J Dent Hyg.

[119] Dishman K, Coan L. Dental Hygiene and Occupational Therapy: Working Together to Improve Oral Care. 5th Celebration of Teaching & Learning Symposium; February 25; The University of Southern Indiana2021.

[120] De Jongh A, Van Houtem C, Van Der Schoof M, Resida G, Broers D (2008). Oral health status, treatment needs, and obstacles to dental care among noninstitutionalized children with severe mental disabilities in The Netherlands. Spec Care Dentist.

[121] Koneru A, Sigal MJ. Access to dental care for persons with developmental disabilities in Ontario. J Can Dent Assoc. 2009;75(2). 19267962

[122] Slack-Smith L, Ree M, Leonard H (2010). Oral health and children with an intellectual disability: a focus group study of parent issues and perceptions. Disabil Health J.

[123] Nelson LP, Getzin A, Graham D, Zhou J, Wagle EM, McQuiston J (2011). Unmet dental needs and barriers to care for children with significant special health care needs. Pediatr Dent.

[124] Como DH, Stein Duker LI, Polido JC, Cermak SA. Oral health and autism spectrum disorders: a unique collaboration between dentistry and occupational therapy. Int J Environ Res Public Health 2021;18(1). 10.3390/ijerph18010135 PMC7795681 33375475

[125] Stein Duker LI, Nelson TM, Webb JR (2019). Adapting oral care protocols to support children with sensory sensitivities: Occupational therapy and dentistry. Dental Care for Children with Special Needs: A Clinical Guide.

