# Supplementary material for: Effectiveness of sensory adaptive dental environments to reduce psychophysiology responses of dental anxiety and support positive behaviours in children and young adults with intellectual and developmental disabilities: a systematic review and meta-analyses
Source: BMC Oral Health. 2023 Oct 19;23:769. doi: 10.1186/s12903-023-03445-6 (PMC10585952; doi:10.1186/s12903-023-03445-6)
Supplement: Supplementary file 2 — Additional file 2. Previous systematic review summary. [file 12903_2023_3445_MOESM2_ESM.docx]

### Appendix B - Previous systematic review summary

| **Citation** | **Search end date** | **Research question/s** | **Databases** | **Participant** | **Inclusion and exclusion criteria** | **Limitations** |
| --- | --- | --- | --- | --- | --- | --- |
| Ismail, Tengku Azmi (1) | Not reported | Not reported | SCOPUS, Medline, CINAHL, Dentistry and Oral Sciences. | 6-12 years  Children in general specifically special needs (did not limit to specific disability). | Inclusion   - Multi/sensory environment - Human studies - English - Abstract Available - Adolescent and adult as subject. - No time limitation - RCT, experimental, pre-experimental, cross-sectional, observational   Exclusion   - Non-human - Review - No abstract - Case studies, report, opinion and qualitative | - Does not search references - Date of search unknown - Didn’t specify limitations of outcomes - Unclear search strategy and search terms (no use of Boolean operators and wild cards) - Vague interpretation of results “high” – no numerical analysis from the studies. - Outcome only behaviour - Poor reporting, unable to replicate study. |
| Mac Giolla Phadraig, Asimakopoulou (2) | Not reported | What nonpharmacological patient support techniques (nPSTs) are used, reported, or recommended to use with people with intellectual developmental disorder (IDD) to receive dental treatment?  What active ingredients or behaviour change techniques (BCTs) are present within nPSTs in the dental setting?  What evidence exists for the effectiveness of BCTs for use with people with IDD to receive dental treatment | Medline, PubMed, Embase,  Cochrane Library, Scopus, Cinahl, and Psychinfo (EBSCO)  Citation searching conducted. | Intellectual-developmental disabilities (not limited by age- adults are children) | Inclusion   - Adults or children with ID - Study reports, uses, or recommends nonpharmacological patient support techniques, specific focus on dental clinical environment. - No comparison, usual treatment, waiting list, control, alternative intervention - All outcome measures acceptable - No date restriction. - Clinical Guidelines, Reviews, Intervention studies and Observational studies - No language restriction   Exclusion   - Case reports, case series, opinion, editorials, and qualitative studies | - Difficulty coding nPSTs. Lack of standardized valid and reliable taxonomy limited data extraction. - 23 included studies although only 1 environment study. |
| Bodison and Diane (3) | Studies from Jan 2007- May 2015 | “What  is the effectiveness of occupational therapy interventions  that use specific sensory techniques or sensory environmental  modifications to support function and participation  of children and youth who have SI difficulties?” | PsycINFO,CINAHL, ERIC, Cochrane Database and OT seeker.  References list | Children (2-21 years) with IDD | Inclusion   - peer-reviewed scientific literature published in English. - examine outcomes of a specific sensory technique or a sensory environmental modification - SI difficulties documented pre and post intervention - Outcome of functional performance or participation. - Involved control group= Levels I, II, and III in the evidence hierarchy   Exclusion   - Intervention not meeting Ayres Sensory Integration approach - Published after 2015 or before 2007. - study design was Level IV or V - SI difficulties not clearly documented - Incorrect outcome measures such as physiological measures and SI functions. | - Outcome focused on participation and functional performance - Only 1 article for environmental strategies. - Publication bias. - Only studies with high level of evidence with verified SI difficulties and reported outcomes of functional participation and performance were added. This led to limited studies included. - May not have included search terms that could have produced eligible studies. |

1. Ismail A, Tengku Azmi T, Malek W, Mallineni S. The effect of multisensory-adapted dental environment on children's behavior toward dental treatment: A systematic review. Journal of the Indian Society of Pedodontics and Preventive Dentistry. 2021;39(1):2-8.

2. Mac Giolla Phadraig C, Asimakopoulou K, Daly B, Fleischmann I, Nunn J. Nonpharmacological techniques to support patients with intellectual developmental disorders to receive dental treatment: A systematic review of behavior change techniques. Special Care in Dentistry. 2020;40(1):10-25.

3. Bodison SC, Diane PL. Specific Sensory Techniques and Sensory Environmental Modifications for Children and Youth With Sensory Integration Difficulties: A Systematic Review. AM J OCCUP THER. 2018;72(1):7201190040p1-p11.
