# Supplementary material for: Effectiveness of sensory adaptive dental environments to reduce psychophysiology responses of dental anxiety and support positive behaviours in children and young adults with intellectual and developmental disabilities: a systematic review and meta-analyses
Source: BMC Oral Health. 2023 Oct 19;23:769. doi: 10.1186/s12903-023-03445-6 (PMC10585952; doi:10.1186/s12903-023-03445-6)
Supplement: Supplementary file 3 — Additional file 3. PICO framework. [file 12903_2023_3445_MOESM3_ESM.docx]

### Appendix C - PICO framework

| Population | Children and adolescents (0-24) with intellectual and developmental disabilities |
| --- | --- |
| Intervention | Sensory adapted environment |
| Comparison | Regular dental environment |
| Outcome | - Compliance, cooperation, and participation. - Negative behaviours or psychophysiology responses of dental anxiety |
