# Supplementary material for: Effectiveness of sensory adaptive dental environments to reduce psychophysiology responses of dental anxiety and support positive behaviours in children and young adults with intellectual and developmental disabilities: a systematic review and meta-analyses
Source: BMC Oral Health. 2023 Oct 19;23:769. doi: 10.1186/s12903-023-03445-6 (PMC10585952; doi:10.1186/s12903-023-03445-6)
Supplement: Supplementary file 4 — Additional file 4. Search strategy. [file 12903_2023_3445_MOESM4_ESM.docx]

### Appendix D - Search strategy

H.1 MEDLINE (Ovid) search strategy

- 1. (Child* or adolescen* or teen or youth or young adult or pe?diatric* or preschool or infant*).ti,ab.
  2. Child, Preschool/ or Pediatrics/ or Adolescent/ or Child/ or infant/ or young adult/
  3. 1 or 2
  4. (Developmental disabilit* or intellectual disabilit* or special need* or mental retardation or disabl* or autis* or ADHD or ASD or Cerebral palsy or attention deficit hyperactivity disorder or Down Syndrome or Fragile X Syndrome or Fetal alcohol spectrum disorder).ti,ab.
  5. Attention Deficit Disorder with Hyperactivity/ or Cerebral Palsy/ or Autistic Disorder/ or Disabled Persons/ or Disabled Children/ or child development disorders, pervasive/ or developmental disabilities/ or intellectual disability/ or Down Syndrome/ or Fragile X Syndrome/ or Fetal Alcohol Spectrum Disorders/
  6. 4 or 5
  7. 3 and 6
  8. ((Dental adj3 (sensory adapted environment* or Multi-sensory environment*)) or Snoezelen or SADE).ti,ab.
  9. Health facility environment/ or environment/ or dental offices/ or environmental adaption/ or Environment, Controlled/
  10. 8 or 9
  11. (((Oral or dental) adj (health or intervention or treatment or procedure or hygiene or anxiety)) or behaviour or compliance or physiological or pain or arousal or stress or psychological).ti,ab.
  12. Stress, Psychological/ or Adaptation, Psychological/ or Psychological Distress/ or Arousal/ or Pain Perception/ or Pain/ or Dental Care/ or Dental Anxiety/ or Sensation/ or Patient Compliance/ or "Treatment Adherence and Compliance"/ or Oral Health/ or Oral hygiene/
  13. 11 or 12
  14. 7 and 10 and 13
  15. Sensory Adapted Dental Environments to Enhance Oral Care for Children with Autism Spectrum Disorders: A Randomized Controlled Pilot Study.m_titl.
  16. Impact of sensory adapted dental environment on children with developmental disabilities.m_titl.
  17. Feasibility of a sensory-adapted dental environment for children with autism.m_titl.
  18. Influence of Adapted Environment on the Anxiety of Medically Treated Children with.m_titl.
  19. 1 5 or 16 or 17 or 18
  20. 14 and 19

H.2 OT Seeker search strategy

- Search all 5 potential titles in all databases no results.
- Basic search retrieved nil articles such as “oral health” “dental anxiety” “sensory adapted environment”.
- Search terms adapted from logic table no results yeiled.

H.3 The Cochrane Library search strategy

- 1. (Child* or adolescen* or teen or youth or young adult or pe?diatric* or preschool or infant*):ti OR (Child* or adolescen* or teen or youth or young adult or pe?diatric* or preschool or infant*):ab
  2. [mh ^" Child, Preschool"] or [mh ^Pediatrics] or [mh ^Adolescent] or [mh ^Child] or [mh ^infant] or [mh ^"young adult"]
  3. (Developmental disabilit* or intellectual disabilit* or special need* or mental retardation or disabl* or autis* or ADHD or ASD or Cerebral palsy or attention deficit hyperactivity disorder or Down Syndrome or Fragile X Syndrome or Fetal alcohol spectrum disorder):ti OR (Developmental disabilit* or intellectual disabilit* or special need* or mental retardation or disabl* or autis* or ADHD or ASD or Cerebral palsy or attention deficit hyperactivity disorder or Down Syndrome or Fragile X Syndrome or Fetal alcohol spectrum disorder):ab
  4. [mh ^"Disabled Persons"] or [mh ^"Disabled Children"] or [mh “Neurodevelopmental Disorders”] or [mh ^"Down Syndome"] or [mh ^"Fragile X Syndrome"] or [mh ^"Fetal Alcohol Spectrum Disorders"]
  5. (#1 OR #2) AND (#3 OR #4)
  6. ((Dental NEAR/3 (sensory adapted environment* or Multi-sensory environment*)) or Snoezelen or SADE):ti OR ((Dental NEAR/3 (sensory adapted environment* or Multi-sensory environment*)) or Snoezelen or SADE):ab
  7. [mh "Dental Facilities"] OR [mh "Health Facility Environment"] OR [mh "Environment, Controlled"]
  8. #6 OR #7
  9. (((Oral or dental) NEXT (health or hygiene or anxiety)) or behaviour or compliance or physiological or pain or arousal or stress or psychological):ti OR (((Oral or dental) NEXT (health or hygiene or anxiety)) or behaviour or compliance or physiological or pain or arousal or stress or psychological):ab
  10. [mh ^”Stress, Psychological”] OR [mh ^”Adaptation, Psychological”] OR [mh ^”Psychological Distress”] OR [mh ^Arousal] OR [mh “Pain Perception”] OR [mh ^Pain] OR [mh ”Dental Care”] OR [mh ^“Dental Anxiety”] OR [mh ^Sensation] OR [mh ^”Patient Compliance”] OR [mh ^“Treatment Adherence and Compliance”] OR [mh ^”Oral Health”] OR [mh ^”Oral Hygiene”]
  11. #9 or #10
  12. #11 AND #8 AND #5
  13. ("Sensory Adapted Dental Environments to Enhance Oral Care for Children with Autism Spectrum Disorders: A Randomized Controlled Pilot Study"):ti OR ("Impact of sensory adapted dental environment on children with developmental disabilities"):ti OR ("Feasibility of a sensory-adapted dental environment for children with autism"):ti OR ("Influence of Adapted Environment on the Anxiety of Medically Treated Children"):ti
  14. #12 AND #13

H.4 Embase (via OVID) search strategy

1. (Child* or adolescen* or teen or youth or young adult or pe?diatric* or preschool or infant*).ti,ab.
2. preschool child/ or pediatrics/ or adolescent/ or child/ or preschool child/ or school child/ or infant/ or young adult/
3. 1 or 2
4. (Developmental disabilit* or intellectual disabilit* or special need* or mental retardation or disabl* or autis* or ADHD or ASD or Cerebral palsy or attention deficit hyperactivity disorder or Down Syndrome or Fragile X Syndrome or Fetal alcohol spectrum disorder).ti,ab.
5. Disabled Persons/ or autism/ or handicapped child/ or developmental disorder/ or intellectual impairment/ or cerebral palsy/ or attention deficit disorder/ or Down syndrome/ or fragile X syndrome/ or fetal alcohol syndrome/
6. 4 or 5
7. 3 and 6
8. ((Dental adj3 (sensory adapted environment* or Multi-sensory environment*)) or Snoezelen or SADE).ti,ab.
9. health care facility/ or environment/ or dental facility/
10. 8 or 9
11. (((Oral or dental) adj (health or hygiene or anxiety)) or behaviour or compliance or physiological or pain or arousal or stress or psychological).ti,ab.
12. mouth hygiene/ or patient compliance/ or sensation/ or dental anxiety/ or pain/ or nociception/ or arousal/ or psychological adjustment/ or mental stress/
13. 11 or 12
14. 7 and 10 and 13
15. Sensory Adapted Dental Environments to Enhance Oral Care for Children with Autism Spectrum Disorders: A Randomized Controlled Pilot Study.m_titl.
16. Impact of sensory adapted dental environment on children with developmental disabilities.m_titl.
17. Feasibility of a sensory-adapted dental environment for children with autism.m_titl.
18. Influence of Adapted Environment on the Anxiety of Medically Treated Children with.m_titl.
19. 15 or 16 or 17 or 18
20. 14 and 19

H.5 Web of Science search strategy

1. Child* or adolescen* or teen or youth or young adult or pe?diatric* or preschool or infant* (Title) or Child* or adolescen* or teen or youth or young adult or pe?diatric* or preschool or infant* (Abstract)
2. Developmental disabilit* or intellectual disabilit* or special need* or mental retardation or disabl* or autis* or ADHD or ASD or Cerebral palsy or attention deficit hyperactivity disorder or Down Syndrome or Fragile X Syndrome or Fetal alcohol spectrum disorder (Title) or Developmental disabilit* or intellectual disabilit* or special need* or mental retardation or disabl* or autis* or ADHD or ASD or Cerebral palsy or attention deficit hyperactivity disorder or Down Syndrome or Fragile X Syndrome or Fetal alcohol spectrum disorder (Abstract)
3. TI=(Dental "NEAR/3" (sensory adapted environment* or Multi-sensory environment*) or Snoezelen or SADE or SAE ) OR AB=(Dental "NEAR/3" (sensory adapted environment* or Multi-sensory environment*) or Snoezelen or SADE or SAE )
4. ((Oral or dental) "NEAR/1" (health or hygiene or anxiety)) or behaviour or compliance or physiological or pain or arousal or stress or psychological (Title) or ((Oral or dental) "NEAR/1" (health or hygiene or anxiety)) or behaviour or compliance or physiological or pain or arousal or stress or psychological (Abstract)
5. #1 AND #2
6. #3 AND #4 AND #5
7. Sensory Adapted Dental Environments to Enhance Oral Care for Children with Autism Spectrum Disorders: A Randomized Controlled Pilot Study (Title) or Impact of sensory adapted dental environment on children with developmental disabilities (Title) or Feasibility of a sensory-adapted dental environment for children with autism (Title) or Influence of Adapted Environment on the Anxiety of Medically Treated Children (Title)
8. #7 AND #6
