# Supplementary material for: Effectiveness of sensory adaptive dental environments to reduce psychophysiology responses of dental anxiety and support positive behaviours in children and young adults with intellectual and developmental disabilities: a systematic review and meta-analyses
Source: BMC Oral Health. 2023 Oct 19;23:769. doi: 10.1186/s12903-023-03445-6 (PMC10585952; doi:10.1186/s12903-023-03445-6)
Supplement: Supplementary file 5 — Additional file 5. Reasons for exclusion of studies. [file 12903_2023_3445_MOESM5_ESM.docx]

### Appendix E - Reasons for exclusion of studies

| **Author/s and Year Published** | **Reason/s for Exclusion** |
| --- | --- |
| Fallea et al. (2022) | Not clinical trial and no randomisation. |
| Unwin, Powell & Jones. (2022) | Not in a dental setting. |
| Shapiro et al. (1997) | Not in a dental setting. |
| Kopel (1977) | Not a clinical trial. |
| Novakovic et al. (2019) | Exceeded inclusion criteria age group. |
| Mitchell & Gaskin. (2004) | Not a clinical trial. |
| Cermak et al. (2015) | Feasibility study. Inappropriate outcome measure. |
| Aljubour et al. (2022) | Intervention not SADE. |
| Fava & Strauss. (2010) | Not in dental setting. |
| Zighair & Jafar. (2021) | Not IDD population. |
| Shapiro et al. (2007) | Not IDD population |
| Potter, Wetzel & Learman. (2018) | Exceeded inclusion criteria age group. |
| Lahman et al. (2008) | Not IDD population. |
| Ghadimi et al. (2018) | Not IDD population |
| Bagattoni et al. (2018) | Not IDD population. |
| Chen & Hawkins. (2021). | Pre/post design. No randomisation |
| Suresh & George. (2019). | No randomisation. |
| Cajares, Rutledge & Haney (2016) | Involved sedation during treatment. |
| Chen et al. (2012) | Not RCT, no control. |
