# Supplementary material for: Effectiveness of sensory adaptive dental environments to reduce psychophysiology responses of dental anxiety and support positive behaviours in children and young adults with intellectual and developmental disabilities: a systematic review and meta-analyses
Source: BMC Oral Health. 2023 Oct 19;23:769. doi: 10.1186/s12903-023-03445-6 (PMC10585952; doi:10.1186/s12903-023-03445-6)
Supplement: Supplementary file 7 — Additional file 7. Intervention criteria. [file 12903_2023_3445_MOESM7_ESM.docx]

### Appendix G - Intervention criteria

| **Sensory modality** | **Example of modifications** |
| --- | --- |
| Visual | Partially dimmed room, lighting effects, bubble tube, mirror ball, visual distraction, kaleidoscopes, lava lamps, wall images, blackout curtains or reducing fluorescent lights. |
| Auditory | Vibroacoustic, music recording, instruments or sound recordings (e.g waves, beach, rain or drums). |
| Tactile | Somatosensory stimuli, deep pressure (compression), tactile objects (e.g squeeze balls), weighted objects, massage or vibrations. |
| Olfactory | Different aromas, essential oils, or scented candles. |
| Vestibular | Vibroacoustic, movement (spinning, jumping or yoga) or rocking/gliding chairs. |
| Gustation | Candy/food (i.e sweet, bitter, mild or sour tastes). |
| Proprioception | Deep pressure (deep pressure), massage, vibration, somatosensory stimuli or weighted objects. |
| Interception | Somatosensory stimuli (pain and/or temperature) |

Modifications are adapted from several multi-sensory intervention studies (1-4)

1. Stein Duker LI. Adapting oral care protocols to support children with sensory sensitivities: Occupational therapy and dentistry. Dental Care for Children with Special Needs: Springer; 2019. p. 77-98.

2. Kim G, Carrico C, Ivey C, Wunsch PB. Impact of sensory adapted dental environment on children with developmental disabilities. Special Care in Dentistry. 2019;39(2):180-7.

3. Unwin KL, Powell G, Jones CR. The use of Multi-Sensory Environments with autistic children: Exploring the effect of having control of sensory changes. Autism. 2021:13623613211050176.

4. Knight M, Adkison L, Kovach JS. A comparison of multisensory and traditional interventions on inpatient psychiatry and geriatric neuropsychiatry units. Journal of psychosocial nursing and mental health services. 2010;48(1):24-31.
