# Supplementary material for: Effectiveness of sensory adaptive dental environments to reduce psychophysiology responses of dental anxiety and support positive behaviours in children and young adults with intellectual and developmental disabilities: a systematic review and meta-analyses
Source: BMC Oral Health. 2023 Oct 19;23:769. doi: 10.1186/s12903-023-03445-6 (PMC10585952; doi:10.1186/s12903-023-03445-6)
Supplement: Supplementary file 8 — Additional file 8. Meta-analysis results for the effect of SADE vs. RDE on EDA and behavioural outcomes during dental procedures. [file 12903_2023_3445_MOESM8_ESM.docx]

### Appendix H - Meta-analysis results for the effect of SADE vs. RDE on EDA and behavioural outcomes during dental procedures

| **Estimate** | **Standard error** | **Z-value** | **P-value** | **Lower confidence interval** | **Upper confidence interval** | **Test for Heterogeneity** |
| --- | --- | --- | --- | --- | --- | --- |
| *Behaviour Meta-analysis* | | | | | | |
| 0.510 | 0.364 | 1.400 | 0.162 | -0.204 | 1.224 | Q(df =1) = 3.981, p-val = 0.046 |
| *EDA Meta-analysis* | | | | | | |
| -0.656 | 0.179 | -3.659 | 0.0003 | -1.007 | -0.304 | Q(df =1) = 0.301, p-val = 0.583 |
